# A Combination of Membrane Filtration and Raman-Active DNA Ligand Greatly Enhances Sensitivity of SERS-Based Aptasensors for Influenza A Virus

**DOI:** 10.3389/fchem.2022.937180

**Published:** 2022-06-30

**Authors:** Gleb Zhdanov, Ekaterina Nyhrikova, Nadezda Meshcheryakova, Olga Kristavchuk, Assel Akhmetova, Evgeny Andreev, Elena Rudakova, Alexandra Gambaryan, Igor Yaminsky, Andrey Aralov, Vladimir Kukushkin, Elena Zavyalova

**Affiliations:** ^1^ Chemistry Department, Lomonosov Moscow State University, Moscow, Russia; ^2^ Joint Institute for Nuclear Research, Dubna, Russia; ^3^ Belozersky Research Institute of Physical Chemical Biology, Lomonosov Moscow State University, Moscow, Russia; ^4^ Institute of Physiologically Active Compounds of Russian Academy of Science, Chernogolovka, Russia; ^5^ Chumakov Federal Scientific Centre for Research and Development of Immune and Biological Products RAS, Moscow, Russia; ^6^ Physical Department, Lomonosov Moscow State University, Moscow, Russia; ^7^ Shemyakin-Ovchinnikov Institute of Bioorganic Chemistry, Russian Academy of Sciences, Moscow, Russia; ^8^ Institute of Solid State Physics, Russian Academy of Science, Chernogolovka, Russia

**Keywords:** aptamer, sensor, membrane, colloidal nanoparticle, SERS, influenza, virus

## Abstract

Biosensors combining the ultrahigh sensitivity of surface-enhanced Raman scattering (SERS) and the specificity of nucleic acid aptamers have recently drawn attention in the detection of respiratory viruses. The most sensitive SERS-based aptasensors allow determining as low as 10^4^ virus particles per mL that is 100-fold lower than any antibody-based lateral flow tests but 10–100-times higher than a routine polymerase chain reaction with reversed transcription (RT-PCR). Sensitivity of RT-PCR has not been achieved in SERS-based aptasensors despite the usage of sophisticated SERS-active substrates. Here, we proposed a novel design of a SERS-based aptasensor with the limit of detection of just 10^3^ particles per ml of the influenza A virus that approaches closely to RT-PCR sensitivity. The sensor utilizes silver nanoparticles with the simplest preparation instead of sophisticated SERS-active surfaces. The analytical signal is provided by a unique Raman-active dye that competes with the virus for the binding to the G-quadruplex core of the aptamer. The aptasensor functions even with aliquots of the biological fluids due to separation of the off-target molecules by pre-filtration through a polymeric membrane. The aptasensor detects influenza viruses in the range of 1·10^3^–5·10^10^ virus particles per ml.

## 1 Introduction

Rapid and specific detection of respiratory viruses has been a key task in the years 2019–2022 due to the wide spread of SARS-CoV-2 virus among the human population. The pandemic revealed the weak spots of our diagnosticum that provided either high sensitivity or rapidness for virus determination. The gold standard of diagnostics is a polymerase chain reaction with reversed transcription (RT-PCR). It takes several hours but has the limit of detection (LoD) in the range of 100–1,300 copies per ml that is equal to 10^2^–10^3^ viral particles per mL for SARS-CoV-2 (VP/ml) (Yang et al., 2021; Kim et al., 2022). Similar characteristics were described for the detection of influenza viruses by RT-PCR; the LoD was reported to be 300–1,200 copies per mL that is equal to 3·10^2^–1.2·10^3^ VP/ml (Herrmann et al., 2001). To shorten the time of the RT-PCR analysis, loop-mediated isothermal amplification (LAMP) was developed with an analysis time of about 1 h. However, the LoD of LAMP is 10 times higher than RT-PCR (2·10^4^ copies/ml versus 2·10^3^ copies/ml, respectively) for SARS-CoV-2 identification (Yan et al., 2020).

Several rapid antibody-based assays have been introduced into clinical usage with a typical analysis time of 10–20 min and the LoD of 1·10^6^–4·10^8^ VP/ml (Chan et al., 2013; Peters et al., 2013).

Surface-enhanced Raman scattering (SERS) provides both rapidness and sensitivity. The best results for virus detection have been obtained for the combination of SERS and DNA aptamers. Aptamers, like antibodies, act as recognizing molecules but have significantly smaller sizes and can be functionalized site specifically during chemical synthesis for anchoring or introducing reporter molecules (Ambartsumyan et al., 2020).

The most sensitive SERS-based aptasensor was developed by Chen et al. (2020) for the influenza A virus with an LoD of 97 plaque-forming units per ml (pfu/ml). This value can be converted to VP/ml using a coefficient of 100 VP/pfu (Kramberger et al., 2012; McCormick and Mermel, 2021), thus, the LoD is 9.7·10^3^ VP/ml being 10 times higher than the LoD of RT-PCR. The same group designed a multi-target sensor that detects influenza A and SARS-CoV-2 viruses simultaneously (Chen et al., 2022). The LoD of the influenza A virus was greatly diminished to 0.62 hemagglutination units per mL (HAU/mL) which is equal to an LoD of 2.8·10^7^ VP/ml using a conversion coefficient of 4.5·10^7^ HAU/VP (Kramberger et al., 2012). This value is comparable with the LoD of rapid antibody-based sensors. As for the LoD of SARS-CoV-2 virus, it was 0.78 pfu/ml that corresponds to 7.8·10^5^ VP/ml based on the coefficient published by Klimstra et al. (2020). Thus, the reported sensors are inferior in sensitivity to RT-PCR methods.

Our previous efforts produced a SERS-based aptasensor on the solid substrate with an LoD of 5 10^–4^ HAU/ml that corresponds to 8 10^4^ VP/ml (Kukushkin et al., 2019) and a SERS-based aptasensor on the aggregated colloid silver nanoparticles with an LoD of 2 10^5^ VP/ml (Gribanev et al., 2021; Gribanyov et al., 2021; Zavyalova et al., 2021; Zhdanov et al., 2022); both were based on the RHA0385 aptamer to hemagglutinin of the influenza A virus (Shiratori et al., 2014). Despite being less sensitive and inferior in a range of virus determination, the latter is preferred because colloid nanoparticles are much cheaper and easier in the production not requiring sophisticated equipment and silicon substrates. Here, we provided an attempt to increase the sensor sensitivity by pre-filtering to eliminate diverse impurities that affect SERS and using a Raman-active dye that provides the characteristic spectrum even at high concentrations of viruses.

## 2 Materials and Methods

### 2.1 Materials

Inorganic salts and tris were purchased from AppliChem GmbH (Darmstadt, Germany). The reagents from Sigma-Aldrich (St. Louis, MO, United States) were used for nanoparticle synthesis, including silver nitrate (AgNO_3_, CAS 7761-88-8), hydroxylamine hydrochloride (NH_2_OH-HCl, CAS 5470-11-1) of the highest purity available, sodium hydroxide (NaOH, CAS 1310-73-2), and sodium chloride (NaCl, CAS 7647-14-5) of analytical-grade purity. Thiolated oligonucleotides were synthesized by Synthol (Moscow, Russia), biotinylated oligonucleotides were synthesized by Evrogene (Moscow, Russia), and oligonucleotides with BODIPY FL were synthesized by Lumiprobe (Moscow, Russia). The sequences were as follows: 1) thiolated RHA0385 aptamer for nanoparticle functionalization aptamer—5′- HS-(CH_2_)_6_-ttggggttattttgggagggcgggggtt-3’; 2) biotinylated RHA0385 aptamer for biolayer interferometry–biotin-5′- ttg​ggg​tta​ttt​tgg​gag​ggc​ggg​ggt​t-3’; and 3) RHA0385 aptamer labeled with the BODIPY FL dye for sandwich-like setup—5′-BODIPY FL-ttggggttattttgggagggcgggggtt-3’. All solutions were prepared using ultrapure water produced with Millipore (Merck Millipore, Burlington, Massachusetts, United States). Buffer A (10 mM tris-HCl pH 7.5, 140 mM NaCl, and 10 mM KCl), buffer B (0.2 mM Tris-HCl pH 7.5, 2.8 mM NaCl, and 0.2 mM KCl), and buffer C (10 mM tris-HCl pH 7.5, 140 mM NaNO_3_, and 10 mM KNO_3_) were used.

### 2.2 Viruses

Influenza viruses and allantoic fluids were provided by the Chumakov Federal Scientific Center for Research and Development of Immune and Biological Products of the Russian Academy of Sciences. The following viruses were studied: influenza A virus A/chicken/Rostock/45/1934 (H7N1) 6^th^ passage (the detailed description is provided by Treshchalina et al. (2021)) and influenza B virus B/Victoria/2/1987; Newcastle disease virus. Viral stocks were propagated in the allantoic cavity of 10-day-old embryonated specific pathogen-free chicken eggs. Eggs were incubated at 37°C, cooled at 4°C for 48 h post-infection, and harvested 16 h later. The study design was approved by the Ethics Committee of the Chumakov Institute of Poliomyelitis and Viral Encephalitides, Moscow, Russia (Approval #4 from 2 December 2014). Viruses were inactivated *via* the addition of 0.05% (v/v) glutaric aldehyde and preserved *via* the addition of 0.03% (w/v) NaN_3_, and stored at +4°C.

The concentration of the viruses was determined in a hemagglutination assay according to Killian (2014). A measure of 50 µl of solutions of the viruses diluted two times step-by-step in a phosphate-buffered saline was placed in a V-shaped 96-well microtiter plate in a volume of 50 µl. Then, 50 µl of 0.5% chicken red blood cells in the phosphate buffered saline was added to the well. The plate was kept in the refrigerator at 4°C for 1 h. Then, the hemagglutination titer was estimated as the maximal dilution of the virus that did not cause the precipitate of red blood cells; this well contained 1 HAU of the virus in the probe.

### 2.3 Preparation of Silver Nanoparticles

Preparation of the silver colloids by reducing a silver nitrate solution with hydroxylamine hydrochloride was conducted according to the method of Leopold and Lendl (2003). The silver nanoparticles were prepared at room temperature under vigorous stirring by a dropwise addition of 10 mM solution of AgNO_3_ (10 ml) to a mixture of 1.67 mM NH_2_OH-HCl with 3.3 mM NaOH (90 ml). The solution was kept under stirring for 1 h after the addition of silver nitrate to ensure a reproducible preparation protocol. A light brown-colored colloidal suspension was obtained with a final pH of 7.0. Before usage of silver nanoparticles for SERS experiments, they were left for 3 months in a refrigerator at +6°–+8°C for aging. The characterization of the nanoparticles with absorption spectroscopy, scanning electron microscopy, dynamic light scattering, and ζ-potential was published in our previous work (Zavyalova et al., 2021). The nanoparticles contained two fractions, namely, 4 and 20 nm, according to scanning electron microscopy. ζ-potential was −55 mV.

### 2.4 Functionalization of Silver Nanoparticles With the Aptamer

The thiolated RHA0385 aptamer was diluted to a concentration of 20 µM in buffer A (for the experiment in Section 3.2. Mechanism of a SERS signal decrease in colloidal aptasensors) or buffer C (for all experiments with BHQ-2 amine). To assemble the G-quadruplex structure of the aptamer, the solution was heated at 95°C for 5 min and cooled to room temperature. An aliquot of 5 µl was added to 5 ml of silver nanoparticles, followed by 30 min of incubation.

### 2.5 Biolayer Interferometry

The biotinylated RHA0385 aptamer was assembled in a concentration of 2 µM in buffer A. The solution was heated at 95°C for 5 min and cooled at room temperature. Biolayer interferometry assays were performed using BLItz (ForteBio, Fremont, CA, United States). Streptavidin biosensors (ForteBio, Fremont, CA, United States) were hydrated in buffer A prior to the experiment.

The protocol for the estimation of RHA0385 aptamer specificity was as follows: 1) the initial baseline in buffer A for 30 s; 2) loading of the aptamer from 2 µM solution in buffer A for 120 s; 3) the second baseline in buffer A with 0.01% (v/v) Tween-20 and 0.1 mg/ml bovine serum albumin for 30 s; 4) the association step in different dilutions of the virus in buffer A with 0.01% (v/v) Tween-20 and 0.1 mg/ml bovine serum albumin for 200 s; 5) the dissociation step in buffer A with 0.01% (v/v) Tween-20 and 0.1 mg/ml bovine serum albumin for 300 s.

The protocol for the estimation of a sandwich-like complex assembly with aggregates of nanoparticles was as follows: 1) the initial baseline in buffer A for 30 s; 2) loading of the aptamer from 2 µM solution in buffer A for 120 s; 3) the second baseline in buffer A for 30 s; 4) the association step in either solution of the influenza A virus and/or aggregates of nanoparticles in buffer A (see the detailed description in the Results section) for 200 s; and 5) the dissociation step in buffer A for 200 s.

The protocol for the estimation of a sandwich-like complex assembly with unaggregated aptamer-functionalized nanoparticles was as follows: 1) the initial baseline in buffer A for 30 s; 2) loading of the aptamer from 2 µM solution in buffer A for 120 s; 3) the second baseline in buffer A for 30 s; 4) the association step in solution of the influenza A virus in buffer A or aptamer-functionalized nanoparticles without the addition of the buffer (see the detailed description in Results section) for 200 s; 5) the third baseline in buffer A for 30 s; 6) the association step in the solution of unaggregated aptamer-functionalized silver nanoparticles for 200 s; and 7) the dissociation step in buffer A for 200 s.

The protocol for the estimation of affinity of BHQ-2 amine to the RHA0385 aptamer was as follows: 1) the initial baseline in buffer A for 30 s; 2) loading of the aptamer from 2 µM solution in buffer A for 120 s; 3) the second baseline in buffer A or buffer B for 30 s; 4) the association step in solutions of BHQ-2 amine in buffer A or buffer B for 200 s; and 5) the dissociation step in buffer A or buffer B for 300 s.

The protocol for the estimation of competition between BHQ-2 amine and the influenza A virus for RHA0385 aptamer binding was as follows: 1) the initial baseline in buffer A for 30 s; 2) loading of the aptamer from 2 µM solution in buffer A for 120 s; 3) the second baseline in buffer B for 30 s; 4) the association step in 1 µM solution of BHQ-2 amine or without BHQ-2 amine in buffer B for 200 s; 5) the third baseline in buffer A for 30 s; 6) association step in solution of influenza A virus in buffer A for 120 s; and 7) the dissociation step in buffer A for 200 s.

The measurements were carried out in black tubes (Sigma-Aldrich, New York, NY, United States) with at least 220 µl of a respective solution in two-three repeats. The curves were approximated exponentially using a 1:1 binding model using exponential curves, according to the guidelines for surface plasmon resonance (Masson et al., 2000). The representation of all binding curves in the logarithmic scale is provided in Supplementary Figures S1–S5, S45.

### 2.6 Sandwich-Like Assay and Characterization of Aggregated Nanoparticles in the Presence of Viruses

Sandwich assay was performed similar to our previous work (Zhdanov et al., 2022). In particular, functionalization of silver nanoparticles with the aptamer was performed in the following way. A measure of 300 µl of viruses (influenza A virus, influenza B virus, Newcastle disease virus or allantoic fluid) diluted in phosphate-buffered saline was incubated with 67 nM of the BODIPY FL-labeled aptamer (concentration of this oligonucleotide was two times higher than the previous setup) (Zhdanov et al., 2022). The solution was mixed with 200 µl of functionalized silver nanoparticles and incubated for 1 min (the time was decreased 4 times compared to our previous work) (Zhdanov et al., 2022). SERS was acquired using a RaPort instrument (Enhanced Spectrometry, United States) with a laser radiation wavelength of 532 nm, and an operating power of 30 mW was used for registration. The spectrometer had a spectral range of 160–4,000 cm^−1^. Spectra were recorded with exposures of 2 s in 10 repetitions. The diameter of the laser beam is 20 microns. The spectrometer slit was 20 microns. The spectrometer scheme is based on the Czerny–Turner design. The laser beam was focused on the center of a 1.5-ml glass vial (Akvilon, Russia), and the scattered light entered the spectrometer back through the same outlet. The same sample preparation was used to study the aggregates of silver nanoparticles in the presence of different viruses. The absorption spectra were acquired with the NanoDrop 2000 spectrometer (Thermo Scientific, Waltham, MA, United States). The sizes of the nanoparticles and aggregates were estimated by dynamic light scattering using Zetasizer Nano ZS (Malvern, Worcestershire, United Kingdom). A total of 3–5 repeats were averaged. Volume distributions were used for further analysis.

### 2.7 Synthesis and SERS Spectra of BHQ-2 Amine

The synthesis of the BHQ-2 amine dye was performed according to the literature (Zavyalova et al., 2022); for the BHQ-2 amine structure Supplementary Figure S7 can be referred. The compound is a derivative of Black Hole Quencher-2 (BHQ-2) with two additional amino groups.

The SERS spectra of BHQ-2-amine were obtained by adding 0.4 µl of 10 µM water solution of the compound to 200 µl of unmodified or aptamer-functionalized silver nanoparticles. After 5-min incubation, the nanoparticles were aggregated by the addition of buffer C or an allantoic fluid (the specific conditions are indicated in the Results section). SERS was acquired using a RaPort instrument (Enhanced Spectrometry, United States) with a laser radiation wavelength of 532 nm, and an operating power of 30 mW was used for registration. Spectra were recorded with exposures of 400 ms in 20 repetitions. The diameter of the laser beam was 20 microns. The spectrometer slit was 20 microns. The spectrometer scheme is based on the Czerny–Turner design. The laser beam was focused on the center of a 1.5-ml glass vial (Akvilon, Russia), and the scattered light entered the spectrometer back through the same outlet.

The wavelength of the laser radiation of 532 nm was chosen based on the following considerations:

1. The BHQ-amine chromophore has an absorption at its maximum (therefore, the effect of resonant Raman scattering is observed) near the wavelength of 532 nm.
2. There is no background signal of the photoluminescence of proteins and DNA fragments in the optical response.
3. The spectrometer is portable and ergonomic due to the use of a semiconductor laser source with a wavelength of 532 nm.
4. The sensitivity of silicon matrices in the spectral range of the device is at its maximum of 540–660 nm.
5. This wavelength excites surface plasmons in silver nanoparticles, and therefore, the SERRS (surface-enhanced resonance Raman scattering) effect is realized on the BHQ-amine substance.

### 2.8 Characterization of Membranes for Virus Filtration

Centrifugal filtration was used to remove non-specific molecules to increase the sensitivity of the SERS-based sensor. Several polyethylene terephthalate track-etched membranes (TM) with different pore diameters were tested as filter elements. The membranes were produced and characterized according to previous works (Flerov et al., 1989; Apel, 1995). The key membrane parameters are shown in Table 1. Track-etched membranes have a strictly calibrated structure and pore size; therefore, they can be used to separate large interfering organic or inorganic particles from the analyte solution. Membranes were obtained in the Flerov Laboratory of Nuclear Reactions, Joint Institute for Nuclear Research.

**TABLE 1:** Characteristics of polyethylene terephthalate track-etched membranes with different pore sizes.

Membrane name	Average diameter by scanning electron microscopy, µ	Average diameter by gas permeability, µ	Pore density, cm^−2^	Membrane thickness, µ
TM-1 (side A)	0.7	0.49	7.9⋅10^7^	19
TM-1 (side B)	0.6			
TM-2 (side A)	0.3	0.32	2.6⋅10^8^	19
TM-2 (side B)	0.4			
TM-3 (side A)	0.02	0.08	1.7⋅10^9^	23
TM-3 (side B)	0.05			

### 2.9 Atomic Force Microscopy Experiment

Atomic force microscopy (AFM) studies were performed using the FemtoScan scanning probe microscope (Advanced Technologies Center, www.nanoscopy.net) in the contact mode. CSG01 cantilevers (NIIFP, Zelenograd) with tip radii in the range of 5–10 nm were used. The sample was mixed with distilled water 1:1 and then applied on a freshly split highly oriented pyrolytic graphite, prolonged for 5 min and dried with a blotter.

The typical scan rate was 1–3 Hz/line. The feedback was adjusted in such a manner so as to reduce the error (deflection) signal. The image consisted of 512 × 512 pixels. Experimental data were processed by FemtoScan Online image software (Advanced Technologies Center, www.nanoscopy.net) (Yaminsky et al., 2016; Yaminsky et al., 2018). The software and sample preparation were previously described in the recent publication with accurate virus visualization (Akhmetova and Yaminsky, 2022).

### 2.10 SERS-Based Sensors for Influenza A Virus Determination

A series of dilutions of the influenza A virus, influenza B virus, or allantoic fluid were prepared in buffer C. The membrane was folded into a cone and put into a 1.5-ml tube; 100 µl of the virus-containing solution was added to the membrane, and the tube was centrifuged for 2 min at 2,400 rpm. Then, the membrane was flushed with 100 µl of buffer C. Additionally, 200 µl of buffer C was added to the solution.

A measure of 0.4 µl of 10 µM aqueous solution of the BHQ-2 amine was added to 200 µl of aptamer-functionalized silver nanoparticles. After 5-min incubation, the nanoparticles were aggregated by the addition of a membrane-filtered virus-containing solution (300 µl).

After 5 min of incubation, the SERS spectrum was acquired using a RaPort instrument (Enhanced Spectrometry, United States) with a laser radiation wavelength of 532 nm, and an operating power of 30 mW was used for registration. Spectra were recorded with exposures of 400 ms in 20 repetitions. The diameter of the laser beam was 20 microns. The spectrometer slit was 20 microns. The spectrometer scheme is based on the Czerny–Turner design. The laser beam was focused on the center of a 1.5-ml glass vial (Akvilon, Russia), and the scattered light enters the spectrometer back through the same outlet.

The overall time of the analysis including all sample preparation steps did not exceed 15 min.

## 3 Results

### 3.1 Selectivity of the DNA Aptamer and an Assembly of a Sandwich-Like Complex

Recently, we have demonstrated the efficiency of several sensors based on the RHA0385 aptamer (Kukushkin et al., 2019; Gribanev et al., 2021; Gribanyov et al., 2021; Zhdanov et al., 2022). Here, we provided data about the selectivity of the aptamer toward influenza A virus and assembling of sandwich-like structures to verify the hypothetic model of the sensor. In all the sensors, the sandwich-like structures made up from the thiolated aptamer, influenza A virus, and BODIPY FL-labeled aptamer were supposed to be assembled. Meanwhile, control viruses such as the influenza B virus or Newcastle disease virus were not supposed to be included in the sandwich-like complexes. If this model does not work, the sensors could be improved by repairing one of the inappropriate steps.

**FIGURE 1:**
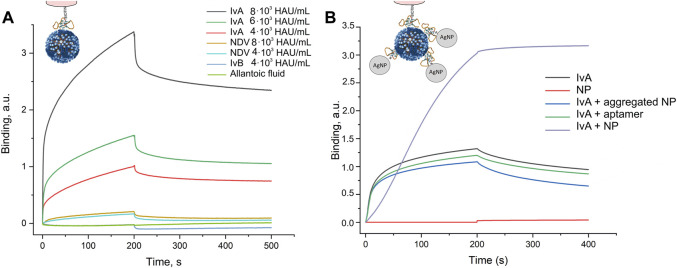
Interaction between the aptamer and viruses assessed by biolayer interferometry. **(A)** Specificity of the aptamer RHA0385 toward the influenza A virus (IvA) compared to the Newcastle disease virus (NDV), influenza B virus (IvB), or virus-free allantoic fluid. The experiments were performed in the buffer with Tween-20 and bovine serum albumin to eliminate non-specific interactions. **(B)** Visualization of an assembly of sandwich-like complexes in the buffer without Tween-20 and bovine serum albumin. The aptamer-functionalized sensor interacts with 1) 4·10^3^ HAU/ml of IvA; 2) aptamer-functionalized silver nanoparticles; 3) 4·10^3^ HAU/ml of IvA with aggregated aptamer-functionalized silver nanoparticles; 4) 4·10^3^ HAU/ml of IvA with a soluble aptamer; and 5) first, with 4·10^3^ HAU/ml of IvA and second, with aptamer-functionalized silver nanoparticles.

Previously, biolayer interferometry was used to estimate the dissociation constant for an aptamer–virus complex; the constant was 0.05 p.m. that corresponds to 0.05 nM recalculating to hemagglutinin concentration (Gribanyov et al., 2021). This value is 10–100 times lower than the dissociation constants for aptamer-recombinant hemagglutinin complexes (Novoseltseva et al., 2020; Bizyaeva et al., 2021). Here, we used the buffer with Tween-20 and bovine serum albumin to eliminate non-specific interactions. This buffer did not allow subtracting the reference sample as its signal is negligible even in the concentrated samples. A new experimental setup revealed that the aptamer RHA0385 provides good selectivity toward the influenza A virus (IvA) compared to the Newcastle disease virus (NDV), influenza B virus (IvB), or a virus-free allantoic fluid (Figure 1A). The virus concentrations were recalculated from hemagglutination units (HAU/ml) to viral particles (VP/ml) using a ratio 1 HAU/ml = 4.5·10^7^ VP/ml, according to Kramberger et al. (2012).The curves were fitted by exponential functions providing an association rate constant of (9 ± 3)·10^8^ M^−1^s^−1^·and a dissociation rate constant of (1.1 ± 0.3)·10^–3^ s^−1^. The equilibrium dissociation constant (K_D_) of the aptamer–virus complex was estimated to be 1.1 ± 0.3 p.m. that it corresponds to K_D_ = 1.1 ± 0.3 nM referring to the monomeric hemagglutinin concentration in the sample (this recalculation is based on the quantity of trimeric hemagglutinin, which is 300–400 times per virus particle (McAuley et al., 2019)). The last value agrees with the dissociation constants of the complexes of the aptamer RHA0385 with recombinant hemagglutinins that varied from 0.6 to 14 nM (Novoseltseva et al., 2020; Bizyaeva et al., 2021).

Next, we tested a possibility of a sandwich-like complex made up from the aptamer anchored on the sensor, influenza A virus, and the aptamer-functionalized silver nanoparticles (Figure 1B). These experiments were performed in the buffer without bovine serum albumin as it non-specifically interacts with silver nanoparticles preventing their aggregation. As was expected, aptamer-functionalized nanoparticles did not interact with the sensor in the absence of the virus. Then, we mixed IvA with aptamer-functionalized nanoparticles in the buffer producing aggregated silver nanoparticles. The binding curve was slightly lower than the curve for the free virus, indicating some competition for virus binding. The competition could be due to the increased concentration of the second aptamer. We tested this hypothesis by mixing the virus with the same amount of the aptamer in the absence of nanoparticles. The resulting curve was lower than the curve from the virus but higher than in the presence of the nanoparticle aggregates. Thus, the aggregates impede the binding of the virus to the sensor *per se*; the aggregates did not bind the sensor over the virus. The next experiment allowed a step-by-step assembly of the complex. The virus was adsorbed from the buffer; next, the sensor with the loaded virus was placed into unaggregated aptamer-functionalized silver nanoparticles. The nanoparticles were absorbed on the sensor with the virus readily. Thus, the sandwich-like complex can be assembled; however, unaggregated nanoparticles are preferable over nanoparticle aggregates.

### 3.2 Mechanism of a SERS Signal Decrease in Colloidal Aptasensors

Our previous colloidal aptasensors used both variants of the sandwich assembly tested in biolayer interferometry. The first one comprises the aggregation of aptamer-functionalized nanoparticles with the labeled aptamer and subsequent addition of the virus sample (Gribanyov et al., 2021); the second one comprises the IvA interaction with the labeled aptamer with subsequent addition of aptamer-functionalized nanoparticles that aggregated in the presence of the virus (Zhdanov et al., 2022). Biolayer interferometry verified the second approach over the first one.

The aptamer was labeled with a fluorescent dye BODIPY FL-RHA0385 that is Raman active; it provided the SERS signal when trapped between several nanoparticles, which was detected with a Raman spectrometer with a 532 nm laser. The SERS signal decreased with an increase in the viral load. Both types of techniques provided a similar limit of detection—2–5·10^5^ VP/ml, whereas the dynamic range of SERS differed: 2·10^5^–2·10^6^ VP/ml for the pre-aggregated nanoparticles (Gribanyov et al., 2021) and 5·10^5^–5·10^7^ VP/ml for the second technique (Zhdanov et al., 2022). The decrease in the SERS-signal is connected with two processes: 1) an interaction between IvA and aptamer-functionalized nanoparticles; 2) a decrease in nanoparticle aggregation in protein-containing solutions. The last point was demonstrated in different dilutions of blood plasma, namely, the SERS signal is absent in 10–100% blood plasma and 5-times lower in 1% blood plasma, being nearly as intensive as in the buffer in 0.1% blood plasma (Zhdanov et al., 2022). The decrease in nanoparticle aggregation can be seen visually as the solution remained yellow-colored instead of gray-colored.

**FIGURE 2:**
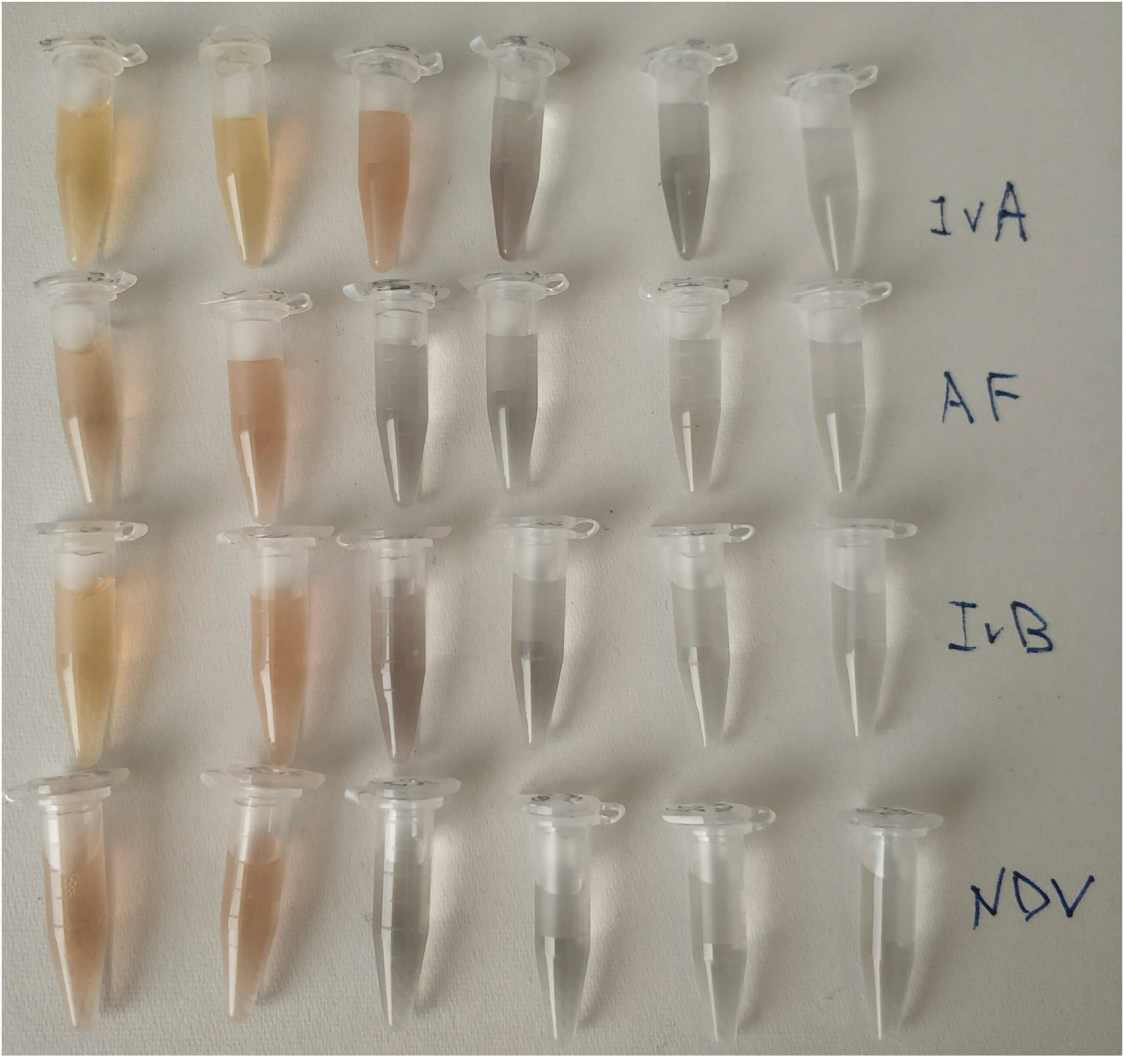
Color changes in the concentrated solutions of viruses. IvA–influenza A virus—from left to right—2·10^10^ VP/ml; 5·10^9^ VP/ml; 1.4·10^9^ VP/ml; 5·10^8^ VP/ml; 1.2·10^8^ VP/ml; and 1.2 10^7^ VP/ml. AF–allantoic fluid–dilutions are the same as for the influenza A virus. IvB–influenza B virus—from left to right—8·10^10^ VP/ml; 2·10^10^ VP/ml; 5·10^9^ VP/ml; 2·10^9^ VP/ml; 5·10^8^ VP/ml; and 5·10^7^ VP/ml; NDV–Newcastle disease—from left to right—4·10^10^ VP/ml; 1.0·10^10^ VP/ml; 3·10^9^ VP/ml; 1·10^9^ VP/ml; 2·10^8^ VP/ml; and 2·10^7^ VP/ml.

**FIGURE 3:**
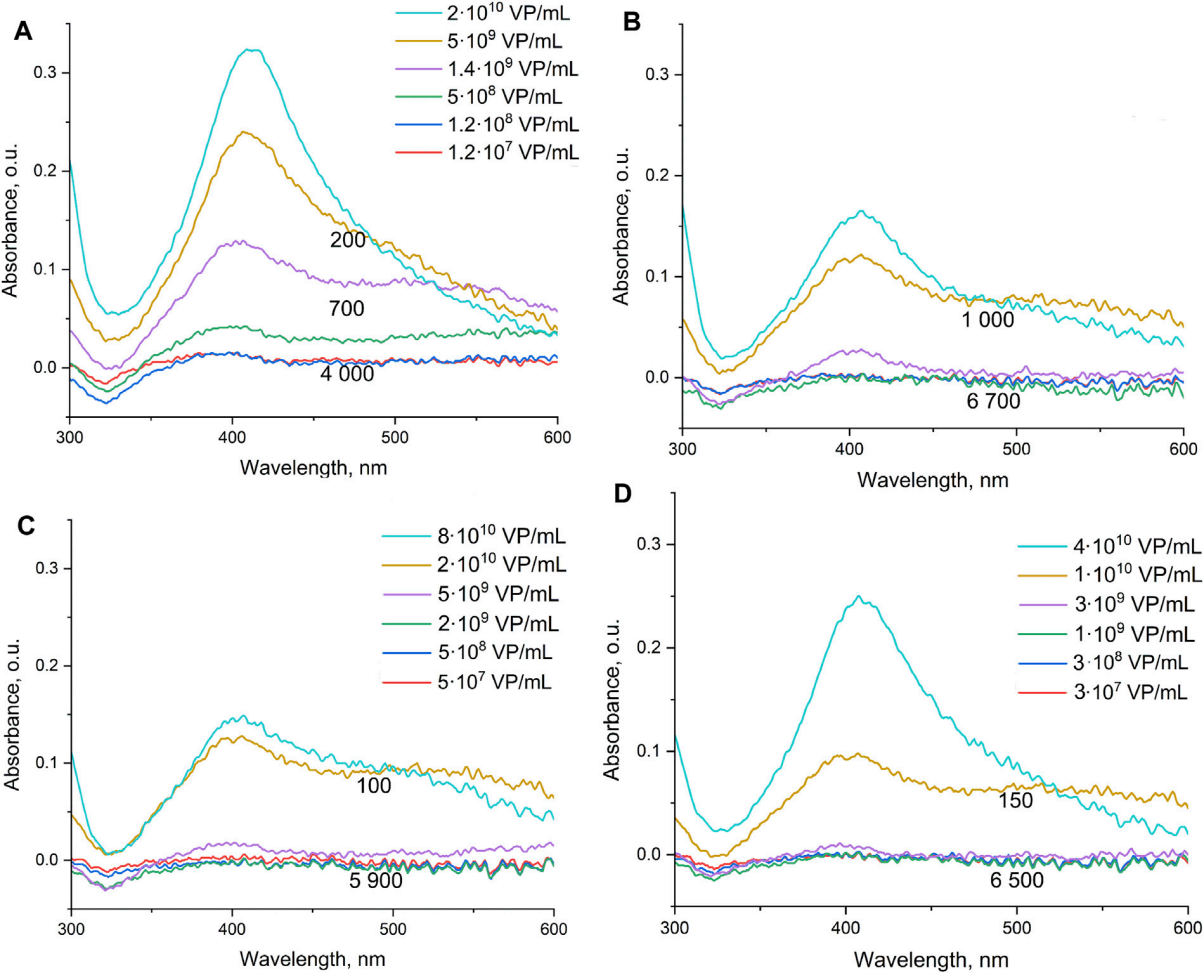
Spectra of samples with different quantities of the influenza A virus **(A)**, allantoic fluid (dilutions are the same as for influenza A virus) **(B)**, influenza B **(C)**, and Newcastle disease virus **(D)**. Numbers indicate the mean SERS intensity of BODIPY FL in the probes. The concentration dependencies were described earlier in Zhdanov et al. (2022).

**FIGURE 4:**
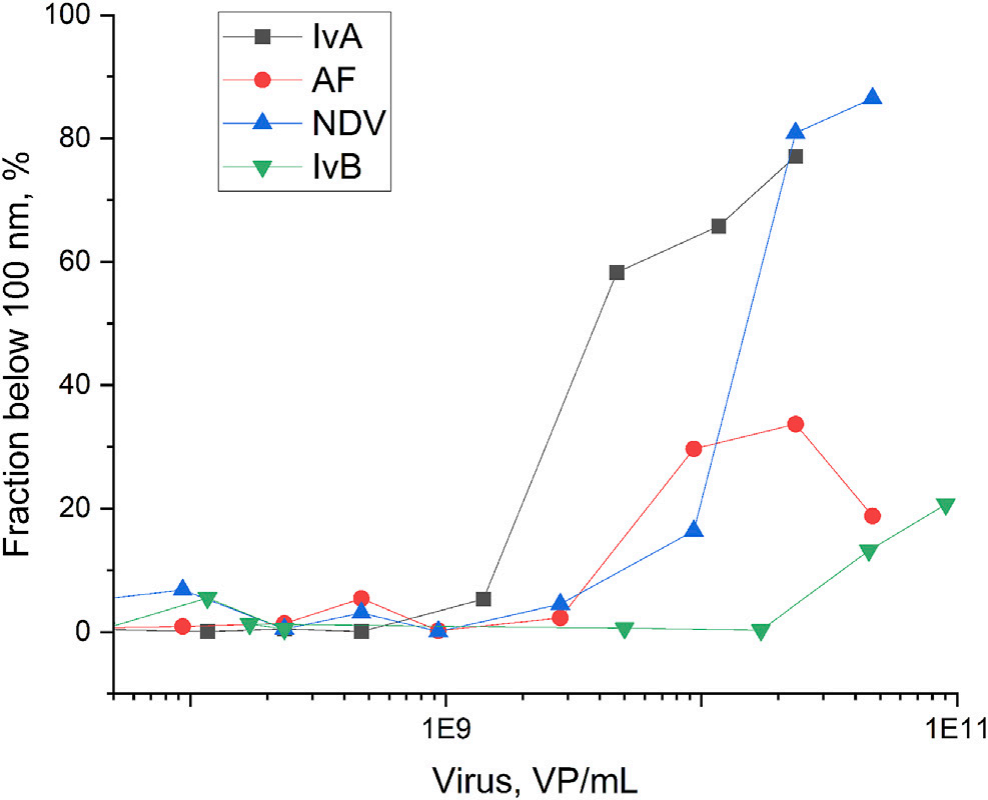
Fraction of the aggregates of silver nanoparticles below 100 nm depending on the viral content. IvA–influenza A virus, IvB–influenza B virus, NDV–Newcastle disease virus, and AF–allantoic fluid. The dilution of allantoic fluid corresponds to the dilution of IvA.

Here, we studied in details the interaction between IvA and aptamer-functionalized nanoparticles. The color changes are provided in Figure 2, and the data on spectrometry are represented in Figure 3. The same solutions were tested in dynamic light scattering to estimate the size of the silver nanoparticle aggregates (Figure 4, Supplementary Figures S7–S43 in Supplementary materials). The yellow color corresponds to small aggregates of the silver nanoparticles with a diameter below 100 nm. The smallest aggregates are larger than the aptamer-functionalized nanoparticles (8 ± 2 nm, see Supplementary Figure S2). Influenza virus increased the fraction of the small aggregates at a lower concentration than other viruses and the allantoic fluid (Figure 4, Supplementary Figures S3–S38). The SERS signal is absent in the yellow solutions of off-target viruses and decreased gradually in gray-to-foggy-to-yellow solutions of the influenza A virus. Disturbance of nanoparticle aggregation by specific and non-specific targets is a main reason of the inability of the aptasensor to produce SERS-signals in biological fluids producing a narrow dynamic range of the sensor. Our further work aimed to overcome this circumstance. First, we used a Raman-active dye that provided signals even in an undiluted allantoic fluid; and second, we used membranes to filter out interfering molecules and macromolecules.

### 3.3 Affinity of BHQ-2 Amine to the DNA Aptamer and Its Raman Spectra on the SERS-Active Surface

**FIGURE 5:**
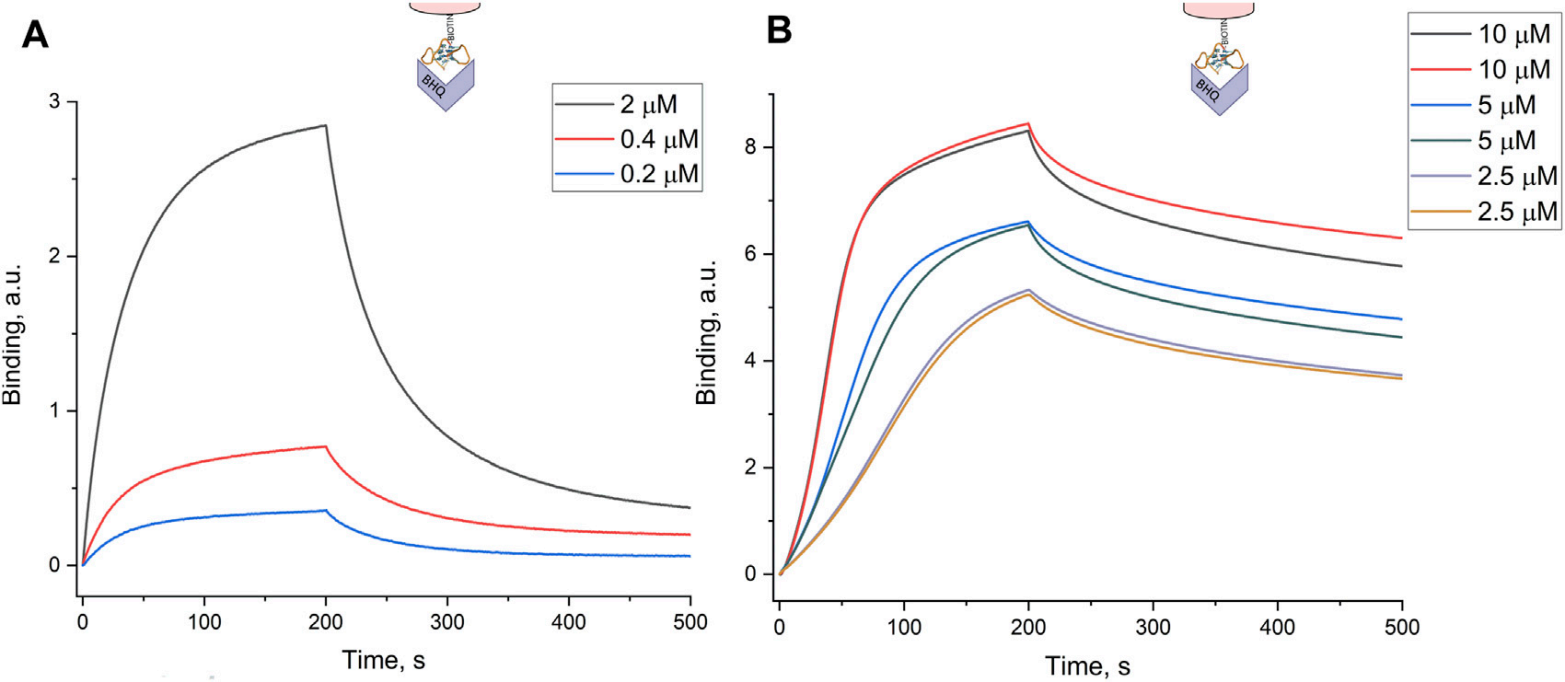
Binding of the BHQ-2 amine to the aptamer RHA0385 estimated by biolayer interferometry. The experiment was performed in **(A)** a buffer with physiological ionic strength (buffer A) and in **(B)** a 50x-diluted buffer (buffer B).

BHQ-2 amine has been developed recently as a Raman-active dye with affinity to non-canonical DNA structures (Zavyalova et al., 2022). It has been shown to bind guanine stacked between G-G and G-A pairs in aptamers for adenosine monophosphate. Here, we used the same dye as a ligand of the guanine quadruplex (G-quadruplex). Aptamer RHA0385 has the structure of parallel G-quadruplex with two G-quartets available for interaction with polyaromatic systems (Novoseltseva et al., 2020). BHQ-2 amine interacts with RHA0385 at physiological ionic strength with dissociation constant of the complex 120 ± 30 nM, whereas a low ionic strength that is used for unaggregated silver nanoparticles increases this value to 680 ± 190 nM (Figure 5; Table 2). These constants are 4–10 times higher than the constant for the original complex of BHQ-2 amine with the aptamer for adenosine monophosphate; however, they are still in the nanomolar range.

**TABLE 2:** Affinity of the BHQ-2 amine to the G-quadruplex aptamer estimated from the data on biolayer interferometry.

Condition	K_D_, nM	k_ass_, mM^−1^s^−1^	k_diss_, ms^−1^
Buffer A	120 ± 30	70 ± 30	7.9 ± 1.7
Buffer B	680 ± 190	1.7 ± 0.6	1.16 ± 0.14

The equilibrium dissociation constants (K_D_), association rate constants (k_ass_), and dissociation rate constants (k_diss_) were estimated in a buffer with physiological ionic strength (buffer A) and in a 50x-diluted buffer (buffer B).

**FIGURE 6:**
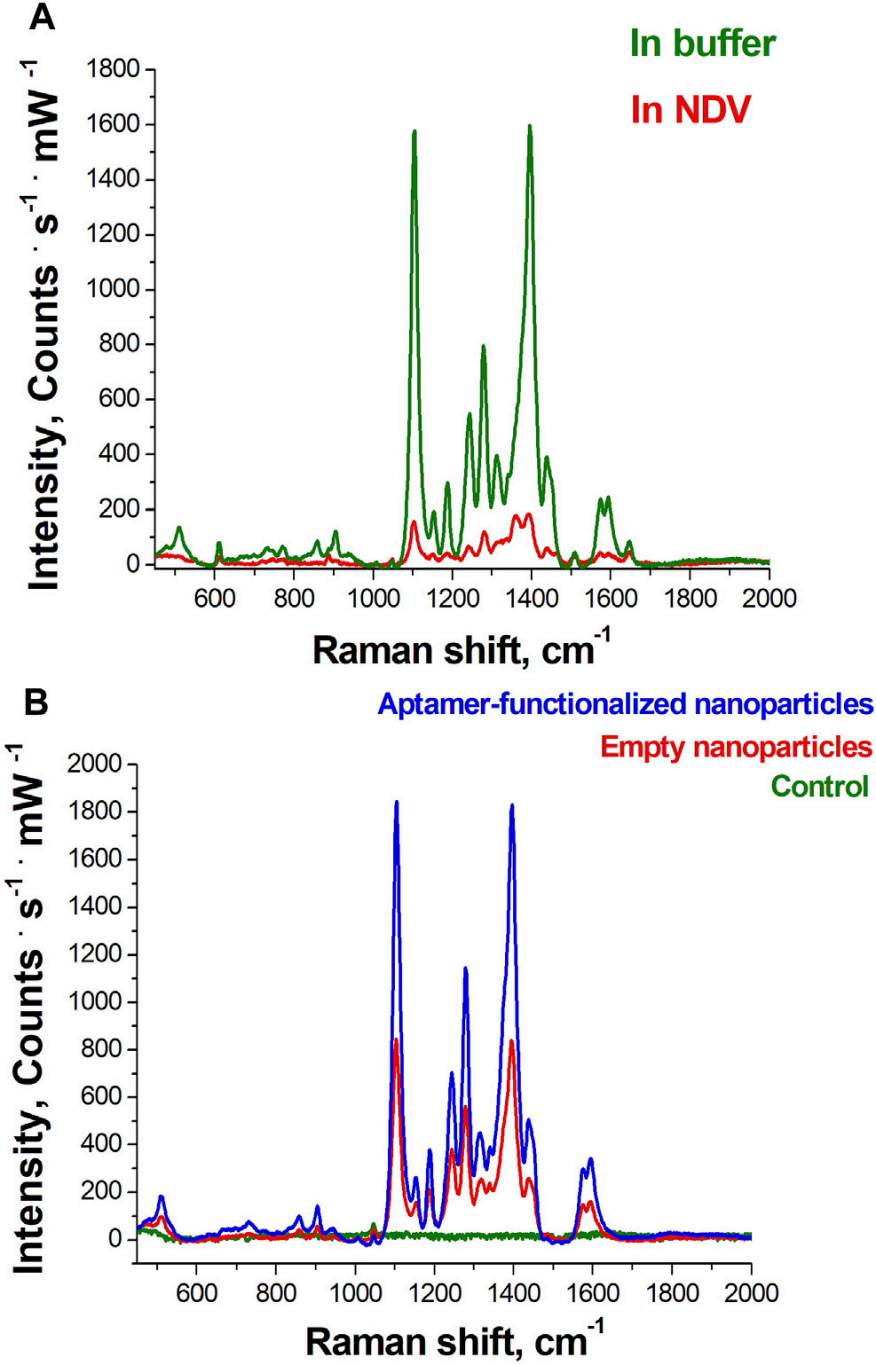
SERS spectra from the BHQ-2 amine in the concentration of 8 nM: **(A)** on aggregated aptamer-functionalized silver nanoparticles in buffer C and in the presence of the Newcastle disease virus (NDV, 10^11^ VP/ml in allantoic fluid); **(B)** on aggregated silver nanoparticles and aggregated aptamer-functionalized silver nanoparticles; green line indicates the Raman spectrum of the BHQ-2 amine in the absence of nanoparticles.

The main advantage of the BHQ-2-amine is its high SERS signal on the colloidal silver nanoparticles. It is diminished 10 times in biological fluids but not disappeared contrary to Cyanine-3 and BODIPY FL dyes that were used in our previous sensors (Figure 6A). The SERS-intensity of the dye was increased two times when the nanoparticles were functionalized with the RHA0385 aptamer (Figure 6B), indicating the assembly of the complex between BHQ-2 amine and the aptamer that increased the local concentration of the dye near the nanoparticle surface.

### 3.4 Competitive Binding of BHQ-2 Amine and Influenza Virus to the DNA Aptamer

**FIGURE 7:**
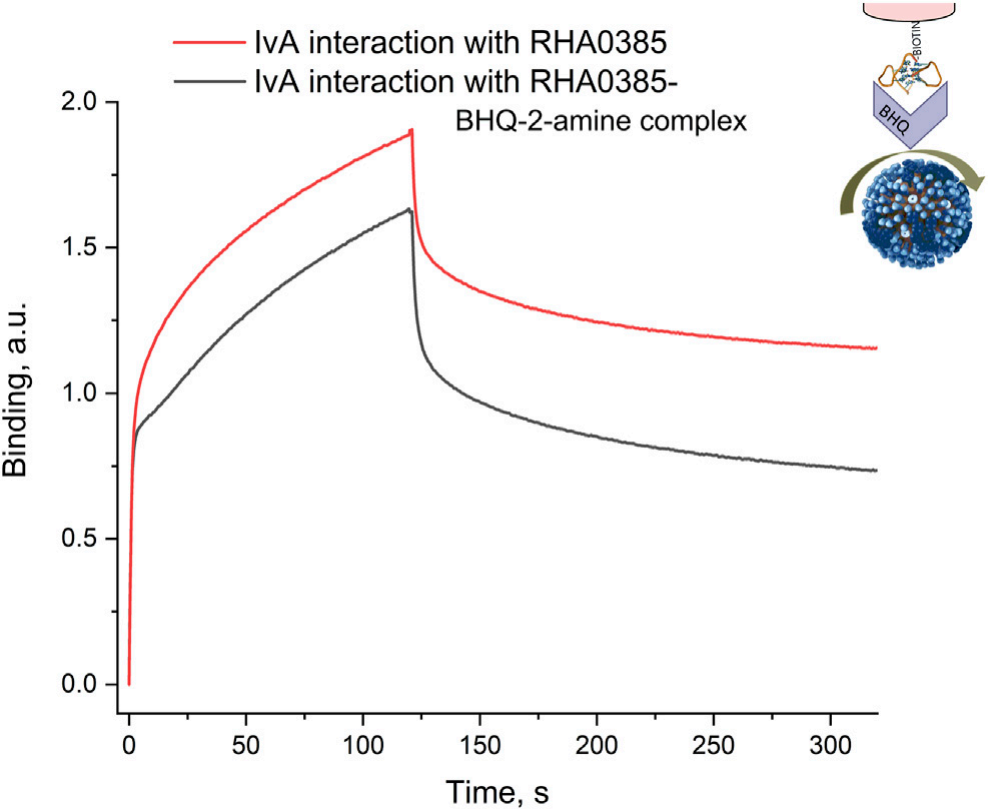
Interaction between the aptamer and its complex with the BHQ-2 amine and the influenza A virus assessed by biolayer interferometry. The aptamer was incubated in the buffer or in a 1 µM solution of the BHQ-2 amine and then interacts with 4·10^3^ HAU/ml solution of IvA.

Analyte-dependent changes in the SERS signal are required to produce sensors based on the BHQ-2 amine. The competition between the BHQ-2-amine and influenza virus for binding to RHA0385 was studied by biolayer interferometry. The aptamer was immobilized onto the sensor and incubated in a buffer without or with 1 µM concentration of the BHQ-2 amine. Then, the sensors were put into solutions of the influenza A virus with a subsequent dissociation step in the buffer. The resultant curves (Figure 7) demonstrated that the interaction between the aptamer and the virus was disrupted if the aptamer was incubated with the BHQ-2 amine. The overall signal is slightly lower, and the dissociation is more pronounced, suggesting that the dye and the virus do compete for aptamer binding.

### 3.5 Usage of Polyethylene Terephthalate Membrane for Virus Filtering

**FIGURE 8:**
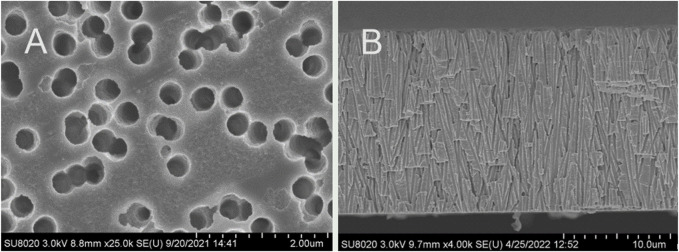
Scanning electron microscopy images of the polyethylene terephthalate membrane with a pore size of 320 nm. **(A)** Front view; **(B)** side view.

The second significant point is to decrease the concentration of low-molecular substances and macromolecules in the sample in order to reduce non-specific interactions. Influenza virus particles are mainly spherical with a characteristic diameter of 100 nm; however, virus particles tend to form aggregates and filamentous structures of 300 nm and higher (Bouvier and Palese, 2008). We tested several polyethylene terephthalate track-etched membranes with pore sizes of 80 nm, 320 nm, and 490 nm. The membrane with a pore size of 80 nm was clogged by the virus, thus hindering filtering. The other two membranes can be used for filtering. The membrane with a medium pore size was chosen for further characterization and development of the sensor. Scanning electron microscopy images revealed the mean outer and inner pore sizes to be of 320 nm (Figure 8). The thickness of the membrane was 19 µ.

**FIGURE 9:**
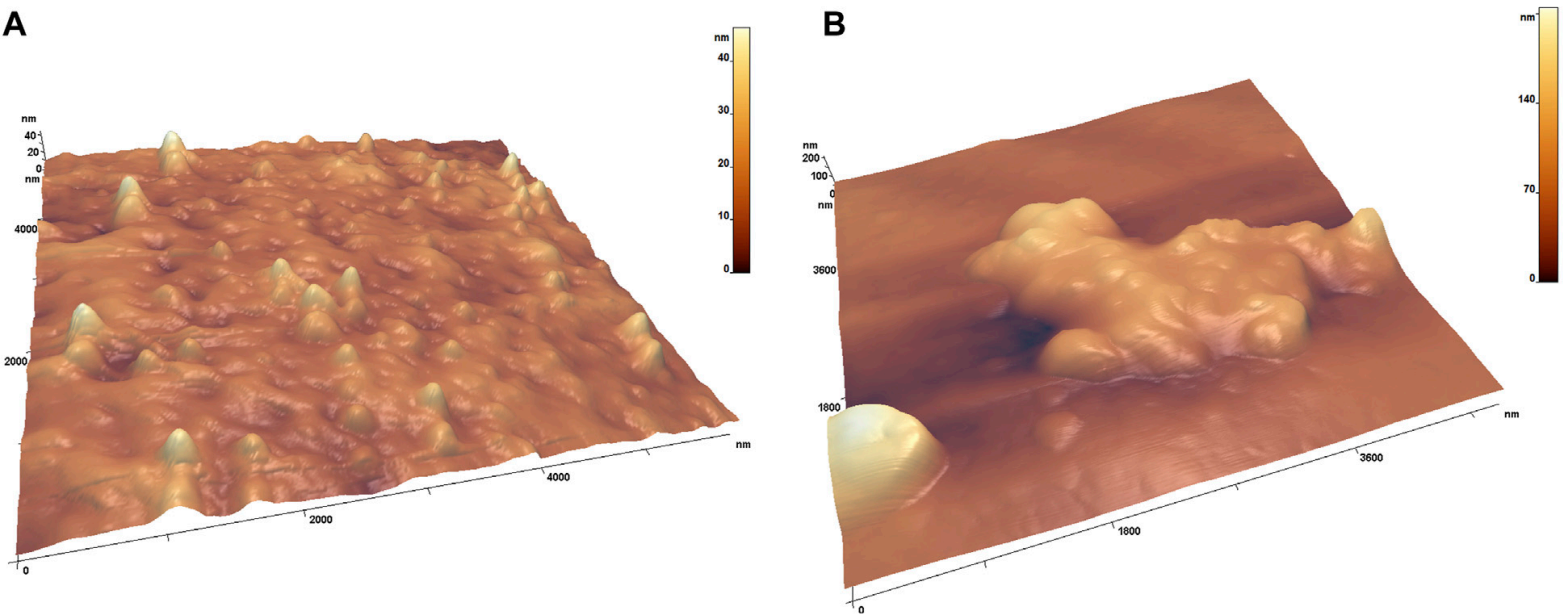
Atomic force microscopy images of the influenza A viruses that passed the membrane **(A)** and were retained by the membrane **(B)**. The maximum height on the A image is in the rate of 30–40 nm; on the B image, it is nearly 180–200 nm.

Next, we estimated the fraction of the virus particles that is held on the membrane during filtering. A measure of 200 µl of the influenza A virus with a concentration of 4,000 HAU/mL was filtered through the membrane by centrifugation. Nearly 200 µL of the solution after filtration was collected, and the membrane was flashed with 200 µL of the buffer to wash out the virus that did not pass the membrane. The virus content in these two fractions was estimated by the hemagglutination assay. The solution after filtration contained 1,000 HAU/ml of the influenza A virus (25%); the membrane flush contained 500 HAU/ml (12.5%), i.e., 62.5% of the virus particles stacked inside the membrane. Thus, the virus concentration in the sample was decreased 8 times; however, the filtration greatly improved the characteristics of the sensor (see the next subsection for details). The virus particles from two fractions were studied by atomic force microscopy, revealing a large amount of aggregates in the fraction that did not pass the membrane and nano-objects of nearly 100 nm in diameter in the filtered fraction (Figure 9). Influenza A viruses tend to form aggregates as hemagglutinin from one viral particle binds to sialic acids on another viral particle (Yen et al., 2011). We suppose this process could be reinforced during filtration through the membrane. Both fractions can bind to the cells, as seen from the hemagglutination assay, and are recognized by the aptamer, as seen from biolayer interferometry (see Supplementary Figures S44, S45).

### 3.6 Characteristics of a SERS-Based Aptasensor for Influenza A Virus Determination With Novel Analyte-Sensitive Dye and Membrane Filtration

**FIGURE 10:**
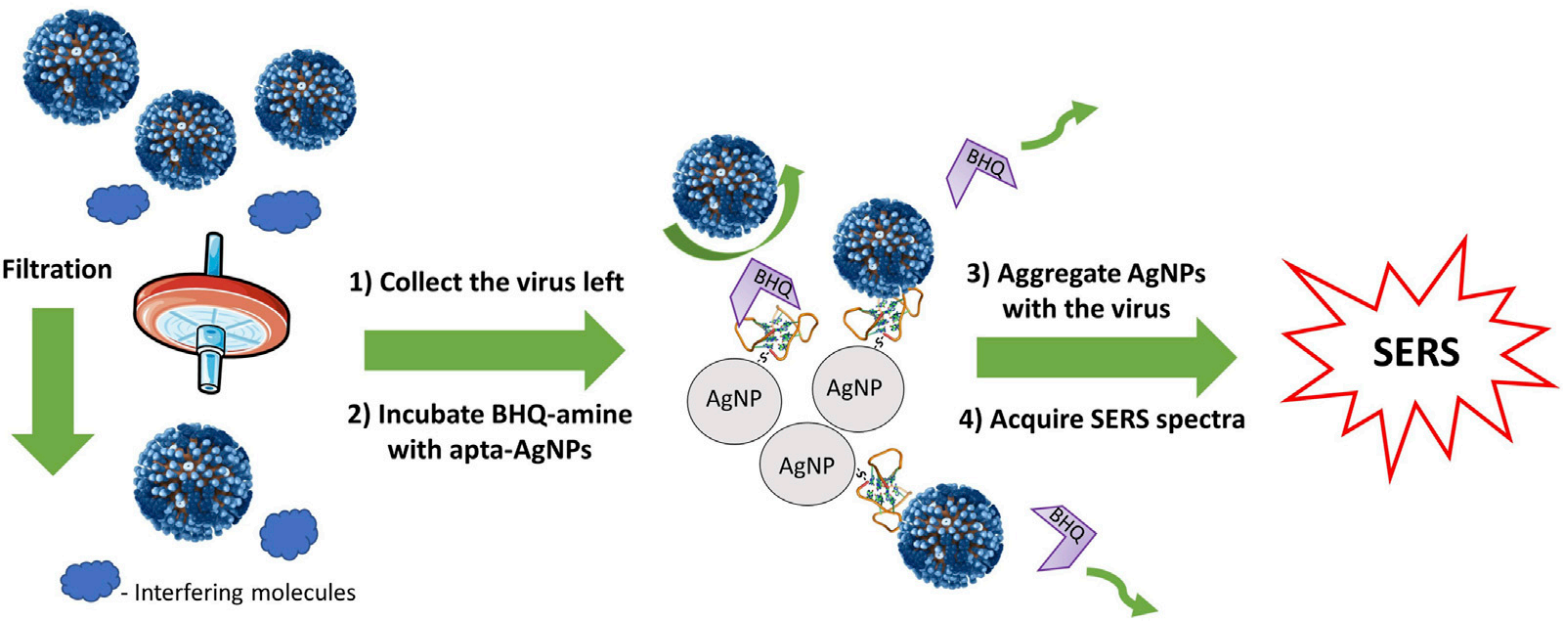
Schematic representation of the proposed sensor. Small objects are filtered out, and large objects displace the BHQ-2 amine from the complex with the aptamer decreasing the SERS signal in the probe.

**FIGURE 11:**
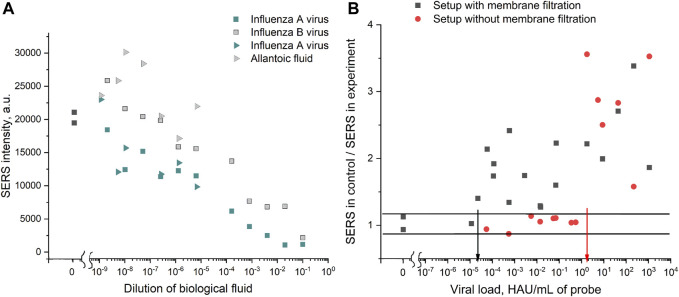
SERS-based aptasensor for influenza A virus. **(A)** Absolute SERS signals for different dilutions of the influenza A virus, influenza B virus, or allantoic fluid. Dilution 10^0^ corresponds to the undiluted sample. The experiments performed simultaneously are shown with the same symbols but in different colors. **(B)** Relative SERS signals obtained by dividing the SERS signal in the control experiment by the SERS signal in the sample with the influenza A virus. Setups with and without membranes are compared. Arrows indicate the limits of detection for each setup. The relative mean standard deviation for the measurement was 15%; thus, the samples with the ratio in the range 0.85–1.15 were interpreted as non-detectable viruses.

The following setup was proposed. Nanoparticles were functionalized with 20 nM of the thiolated aptamer RHA0385, then incubated with 8 nM solution of BHQ-2 amine for 5 min, and aggregated with a membrane-filtered solution of the influenza A virus or a control biological solution (Figure 10). To estimate the contribution of the membrane filtration, an experiment without this step was performed (Figure 11). The SERS signal increased nearly monotonously with a dilution of the biological fluid. Similar results were obtained earlier for SERS in different dilutions of the blood plasma (Zhdanov et al., 2022). However, the signal in samples with the influenza A virus was nearly two times lower than the control biological fluids. This difference was attributed to the competition between the BHQ-2 amine and the virus for aptamer binding. As the aptamer provided a two-fold increase of the SERS signal for BHQ-2 amine (Figure 6B), the two-fold differences could be interpreted as disruption of the aptamer-BHQ-2-amine complex by the virus. The absolute SERS cannot be interpreted directly as it depends on the concentration of macromolecules in the allantoic fluid. Therefore, relative SERS signals were plotted versus a viral load before the membrane filtration (Figure 11B). Notably, the real viral load is significantly lower for the samples with membrane filtration as an estimated fraction of the virus on the membrane was 12.5% for the undiluted virus. This point could be improved by optimizing the efficiency of membrane filtration. Even based on the viral content before the filtration, membrane filtration decreased the limit of detection of the sensor by five orders of the magnitude—from 1.7 HAU/ml to 2.2·10^–5^ HAU/ml (Figure 11B). Thus, membrane filtration is crucial for the sensitivity of the technique.

## 4 Discussion

Surface-enhanced Raman spectroscopy is a highly sensitive technique; however, there is an urgent need for reproducible and cheap SERS-active substrates. The SERS substrates with the highest Raman enhancement coefficient are produced using high technological methods like lithography (Chen et al., 2020; Choi et al., 2020). Colloid silver nanoparticles are very cheap and simple in production; however, their SERS signal is highly dependent on the concentration of various biologicals (Zhdanov et al., 2022). Here, we reduced this effect by employing sample pre-filtering. The efficient decrease in the non-specific biologicals is illustrated in Supplementary Figure S46, where the mixtures of silver nanoparticles with unfiltered samples are compared with the mixtures of silver nanoparticles with filtered samples. A yellow color in unfiltered samples confirms the low efficiency of nanoparticle aggregation, whereas the gray color corresponds to the formation of large aggregates with high Raman enhancement coefficients. The correlation between the color, aggregate size, and SERS intensity of the samples with viruses is shown in Figures 2–4.

Another significant point is to use Raman-active dyes that are efficient even in the presence of biologicals. Recently, we have found that the BHQ-2 amine and the BHQ-2 guanidine provide strong SERS signals compared to classic BHQ-2. The positive charge of the dye was supposed to play the key role in the enhancement of Raman spectra due to adsorption on the surface of negatively charged nanoparticles (Zavyalova et al., 2022). In this work, the BHQ-2 amine was shown to provide SERS spectra even in undiluted biological fluids (Figure 6), whereas other soluble dyes (cyanine 3, Bodipy FL, rhodamine 6G, BHQ-2, and cyanine 5) were not detected under these conditions. The BHQ-2 amine was shown to be a ligand of the G-quadruplex aptamer RHA0385; moreover, it competed with the influenza A virus for aptamer binding. This competitive binding was used successfully for influenza A virus detection with the limit of detection of 2.2·10^–5^ HAU/ml that corresponds to 1,000 VP/ml (Kramberger et al., 2012). Nearly the same LoD of 10^3^ VP/ml was previously shown for RT-PCR (Herrmann et al., 2001) and electrochemical aptasensor (Kiilerich-Pedersen et al., 2013). Further improvement of membrane filtering can decrease the LoD lower than 10^3^ VP/ml as the current filtration retained only 12.5% of the influenza A viruses. A combination of microfluidics and membranes with pore sizes of nearly 100 nm is estimated to be a promising way to provide even more sensitive sensors with an overall time of analysis below 15 min.

Aggregates of silver nanoparticles provide a wide plasmonic peak allowing the use of several laser wavelengths, e.g., 633 nm. Concurrent usage of two or three lasers can be applied to visualize the enhancement inside the nanoparticle aggregates similar to the previous work (Asiala et al., 2015). The aggregates can be also deposited on the transparent substrate providing a solid-state SERS substrate similar to those reported previously (Jayawardhana et al., 2013).

It is important to discuss the possibility of incorporating the SERS-based techniques in a routine laboratory analysis. SERS-based techniques provide high sensitivity with a short time of analysis. We aimed to minimize the time of analysis to 15 min in this work and our previous works (Kukushkin et al., 2019; Gribanev et al., 2021; Gribanyov et al., 2021; Zavyalova et al., 2022; Zhdanov et al., 2022), which places the techniques in the raw of rapid point-of-care tests. SERS equipment is of rather high cost; here, we used a portable device with the price 15,000 USD with a user-friendly interface (RaPort instrument from Enhanced Spectrometry, United States). This equipment minimizes special work skills of the operator training allowing the spectra to be recorded automatically, thereby minimizing human involvement and measurement error. The price of the sensor itself is extremely low, 0.2 USD, compared to the price of the test strips of 20–30 USD. The low price was achieved due to the cheap and simple production of silver colloids, large-scale production of membranes, low concentrations of dyes, and aptamers. The cost of the piece of equipment with large-scale use in clinical practice will pay off quickly enough due to the large difference in cost between the developed sensor and test strips. PCR provides the same sensitivity within several hours with the typical price of the equipment about 40,000 USD and 15–25 USD per sample (the data are given from the WHO official website). Thus, SERS-based techniques can enter laboratory diagnostics in the near future.

## Data Availability

The original contributions presented in the study are included in the article/Supplementary Material; further inquiries can be directed to the corresponding author.
