# Supplementary material for: A Combination of Membrane Filtration and Raman-Active DNA Ligand Greatly Enhances Sensitivity of SERS-Based Aptasensors for Influenza A Virus
Source: Front Chem. 2022 Jun 30;10:937180. doi: 10.3389/fchem.2022.937180 (PMC9279936; doi:10.3389/fchem.2022.937180)
Supplement: Supplementary file 1 [file DataSheet1.PDF]

Supplementary materials for

A combination of membrane filtration and Raman-active DNA ligand greatly enhances sensitivity of SERS-based aptasensors for influenza A virus

Running title: SERS-based aptasensor for influenza virus

Gleb Zhdanov, Ekaterina Nyhrikova, Nadezda Meshcheryakova, Olga Kristavchuk, Assel Akhmetova, Evgeny Andreev, Elena Rudakova, Alexandra Gambaryan, Igor Yaminsky, Andrey Aralov, Vladimir Kukushkin, Elena Zavyalova

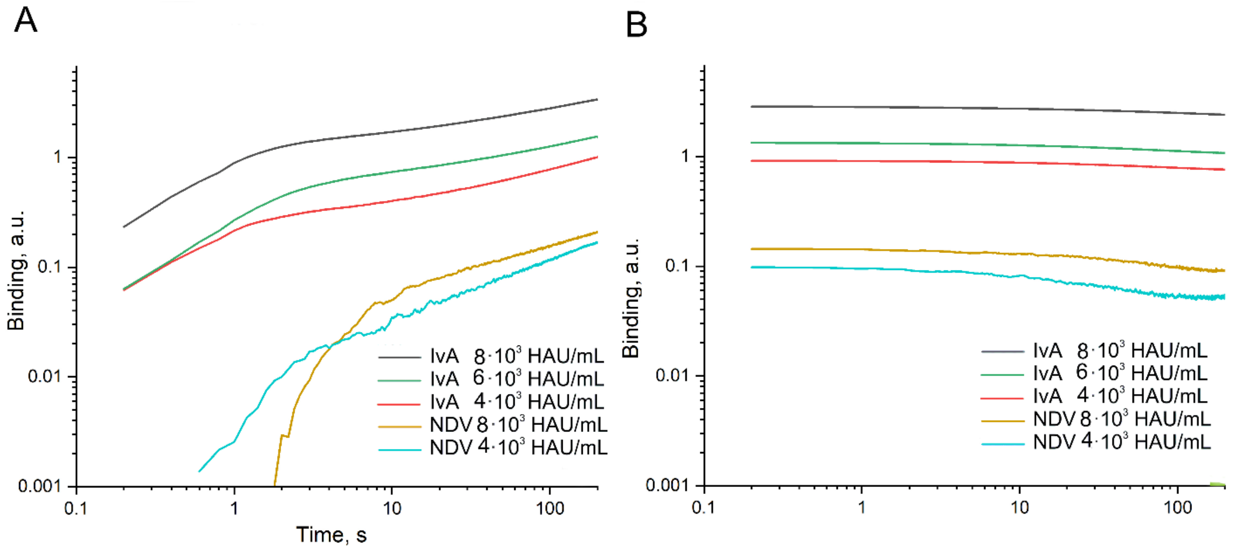

Figure S1. Interaction between the aptamer and viruses assessed by biolayer interferometry. The data are the same as in Figure 1A in the main text but represented in the logarithmic scale. Specificity of aptamer RHA0385 towards influenza A virus (IvA) compared to Newcastle disease virus (NDV). The experiments were performed in the buffer with Tween-20 and bovine serum albumin to eliminate non-specific interactions. The curves for influenza B virus or virus-free allantoic fluid are absent as their binding intensities were below zero.

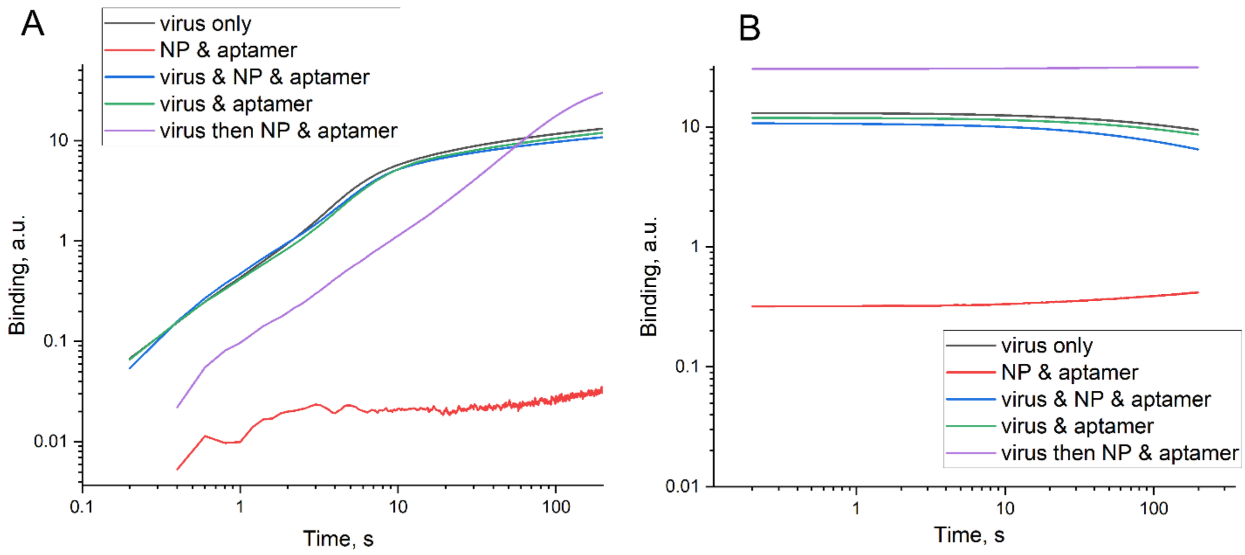

Figure S2. Interaction between the aptamer and viruses assessed by biolayer interferometry. The data are the same as in Figure 1B in the main text but represented in the logarithmic scale. The visualization of an assembly of sandwich-like complexes in the buffer without Tween-20 and bovine serum albumin. The aptamer-functionalized sensor interacts with 1) $4{\cdot}10^3$ HAU/mL of IvA; 2) aptamer-functionalized silver nanoparticles; 3) $4{\cdot}10^3$ HAU/mL of IvA with aggregated aptamer-functionalized silver nanoparticles; 4) $4{\cdot}10^3$ HAU/mL of IvA with soluble aptamer; 5) firstly, with $4{\cdot}10^3$ HAU/mL of IvA and, secondly, with aptamer-functionalized silver nanoparticles.

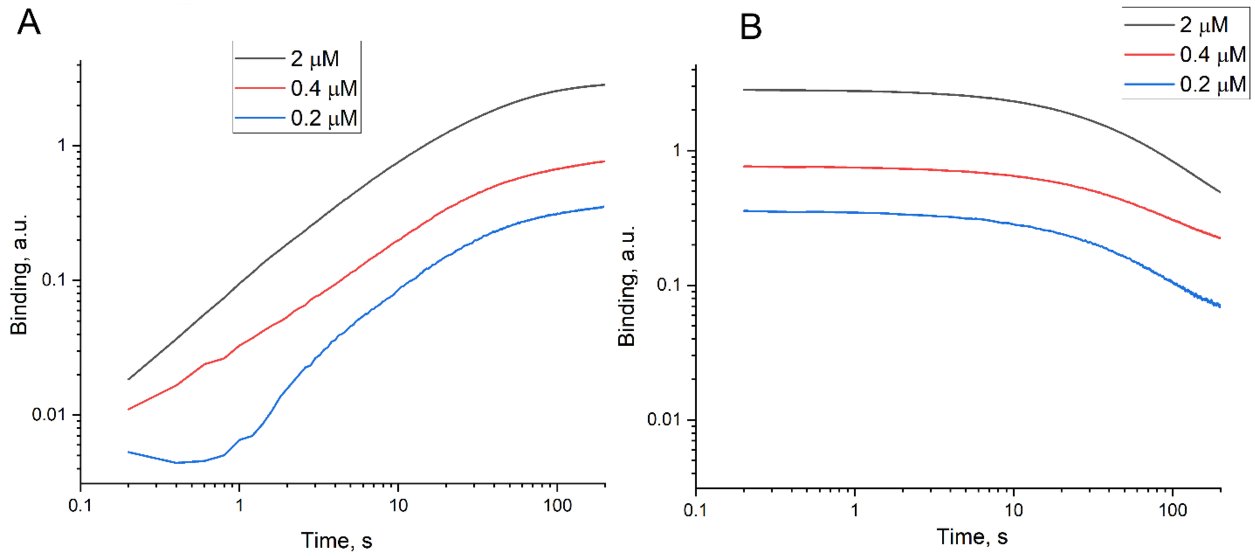

Figure S3. Binding of BHQ-2-amine to aptamer RHA0385 estimated by biolayer interferometry. The data are the same as in Figure 5A in the main text but represented in the logarithmic scale. The experiment was performed in a buffer with physiological ionic strength (Buffer A).

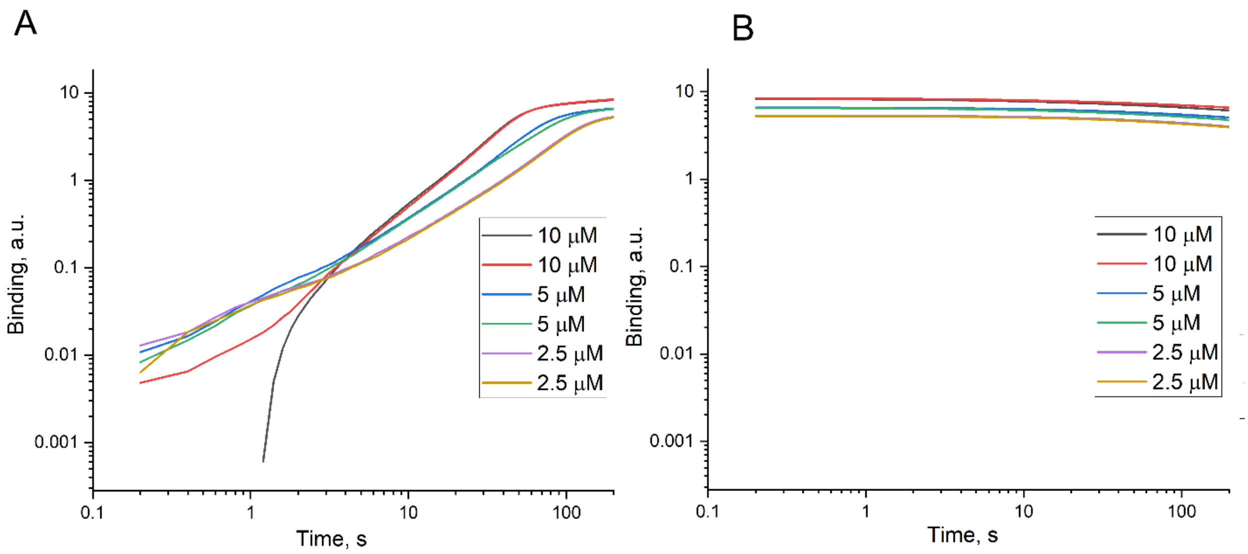

Figure S4. Binding of BHQ-2-amine to aptamer RHA0385 estimated by biolayer interferometry. The data are the same as in Figure 5B in the main text but represented in the logarithmic scale. The experiment was performed in a 50x-diluted buffer (Buffer B).

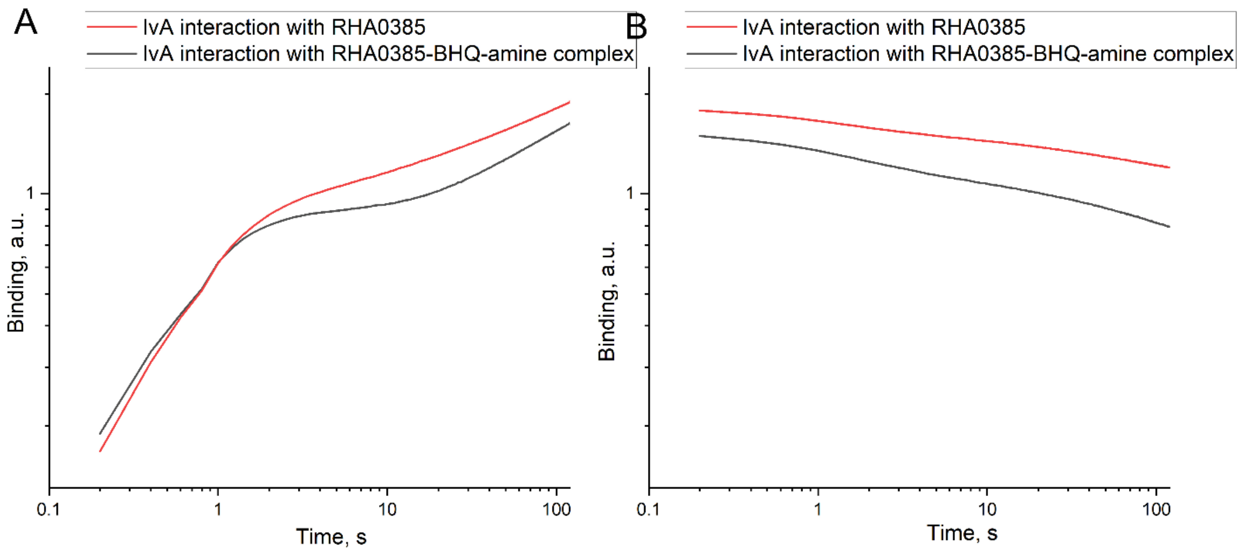

Figure S5. Interaction between the aptamer or its complex with BHQ-2-amine and influenza A virus assessed by biolayer interferometry. The data are the same as in Figure 7 in the main text but represented in the logarithmic scale. The aptamer was incubated in the buffer or in 1 µM solution of BHQ-2-amine and then interacts with $4\cdot10^3$ HAU/mL solution of IvA.

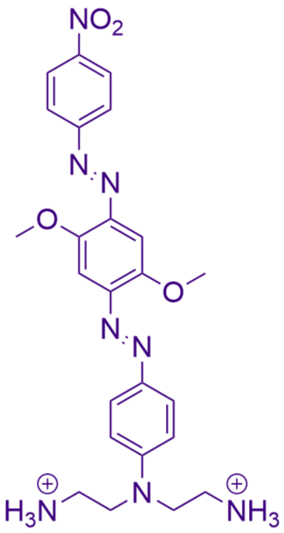

Figure S6. Chemical formulae of BHQ-amine.

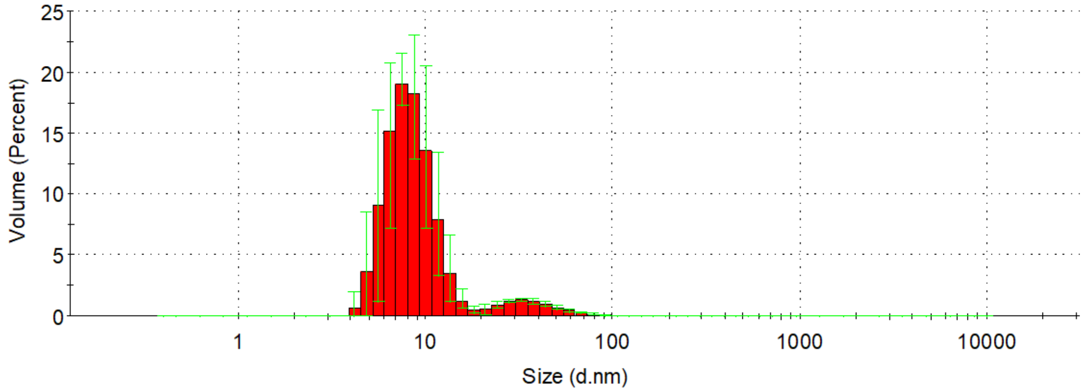

Figure S7. Dynamic light scattering. The volume distribution of silver nanoparticles.

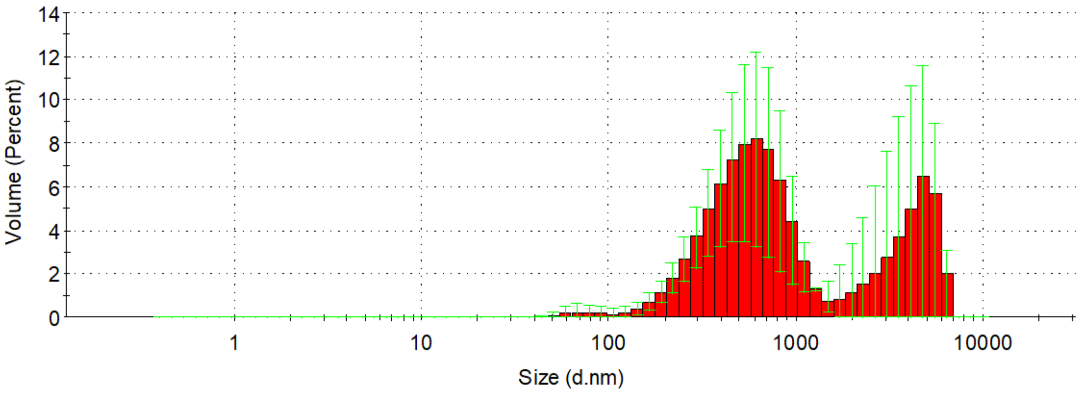

Figure S8. Dynamic light scattering. The volume distribution of silver nanoparticles in the sensor with $1.2 \cdot 10^7$ VP/mL of influenza A virus.

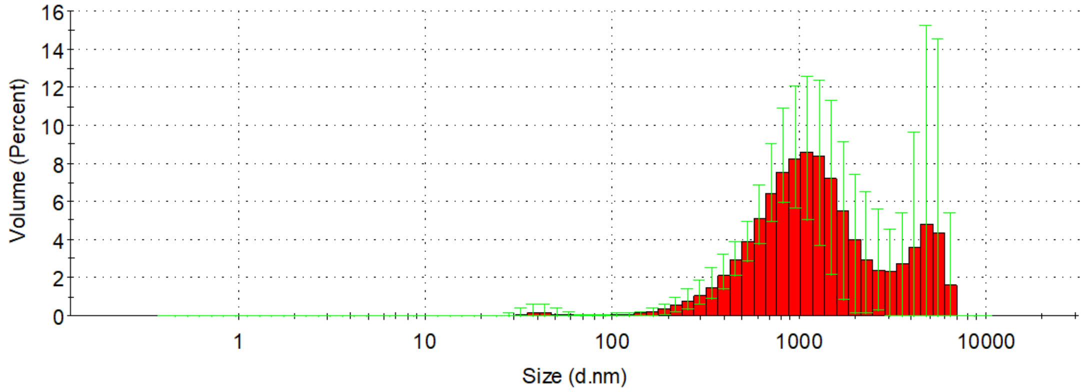

Figure S9. Dynamic light scattering. The volume distribution of silver nanoparticles in the sensor with $5 \cdot 10^{7}$ VP/mL of influenza A virus.

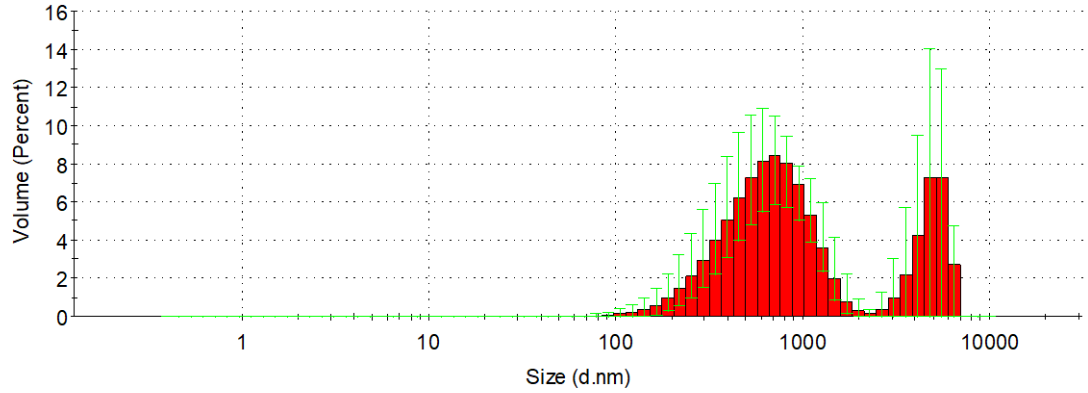

Figure S10. Dynamic light scattering. The volume distribution of silver nanoparticles in the sensor with $1.2 \cdot 10^{8}$ VP/mL of influenza A virus.

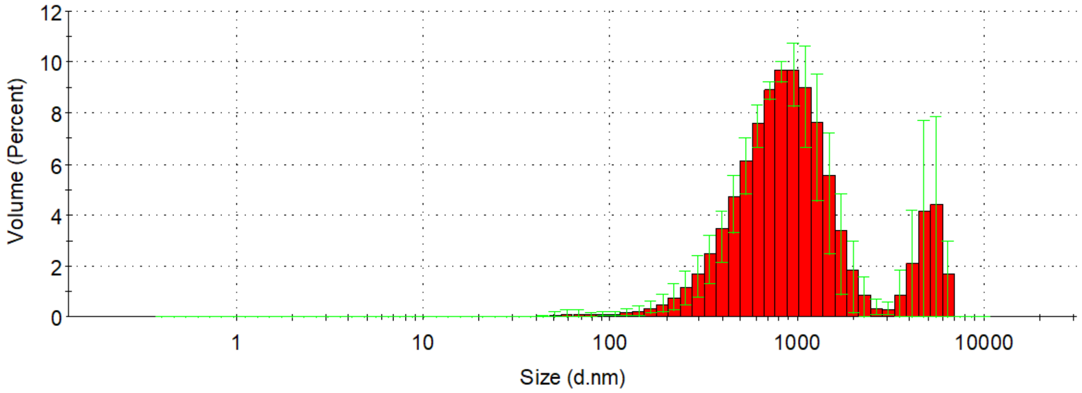

Figure S11. Dynamic light scattering. The volume distribution of silver nanoparticles in the sensor with $2.5 \cdot 10^{8}$ VP/mL of influenza A virus.

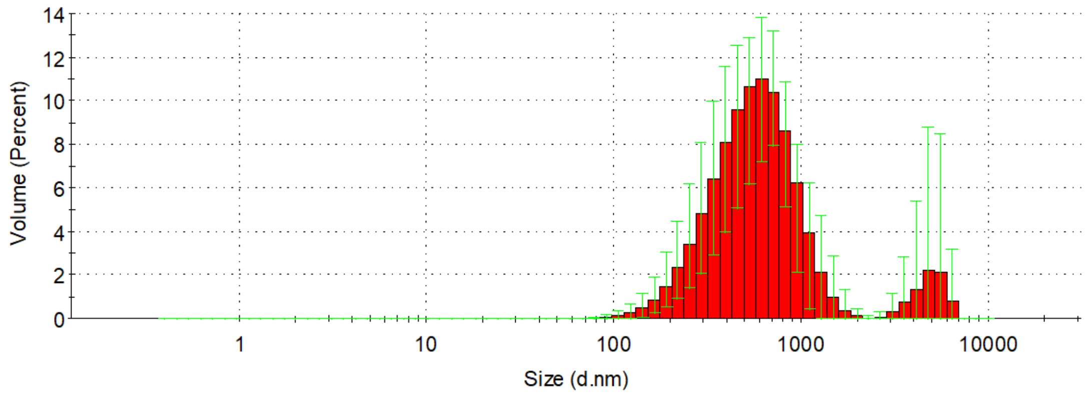

Figure S12. Dynamic light scattering. The volume distribution of silver nanoparticles in the sensor with $5 \cdot 10^8$ VP/mL of influenza A virus.

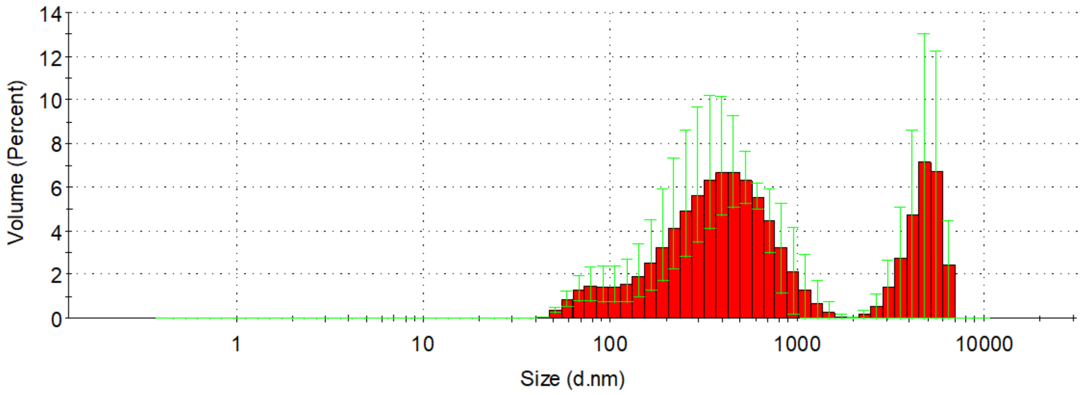

Figure S13. Dynamic light scattering. The volume distribution of silver nanoparticles in the sensor with $1.4 \cdot 10^9$ VP/mL of influenza A virus.

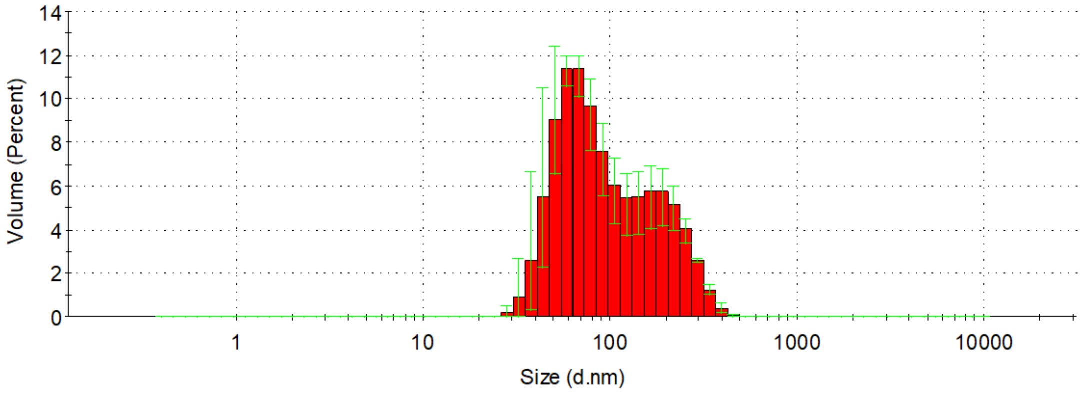

Figure S14. Dynamic light scattering. The volume distribution of silver nanoparticles in the sensor with $5 \cdot 10^9$ VP/mL of influenza A virus.

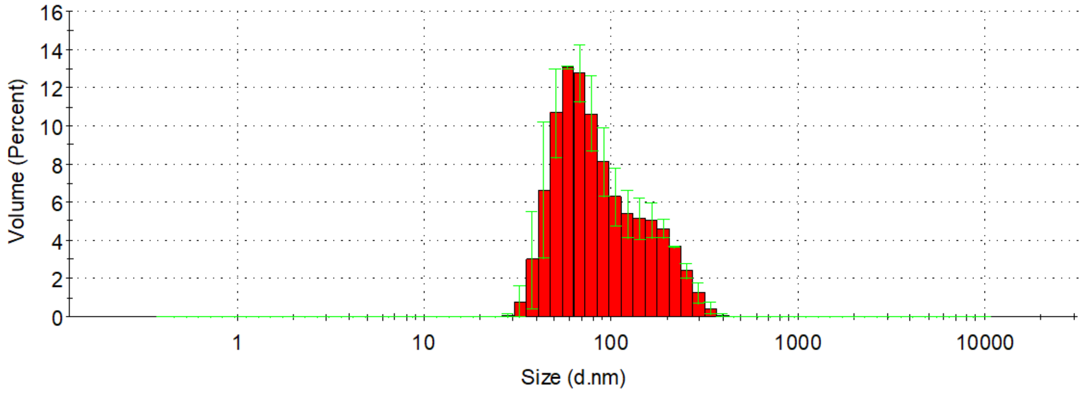

Figure S15. Dynamic light scattering. The volume distribution of silver nanoparticles in the sensor with $1{\cdot}10^{10}$ VP/mL of influenza A virus.

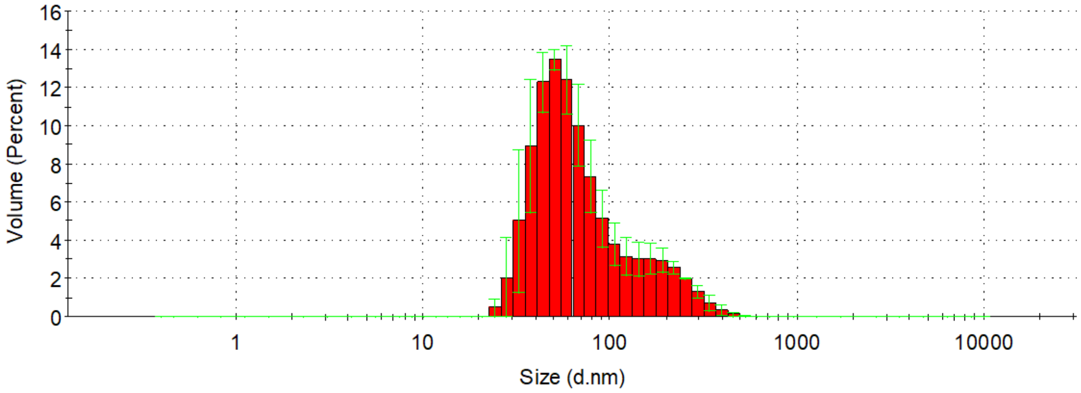

Figure S16. Dynamic light scattering. The volume distribution of silver nanoparticles in the sensor with $2{\cdot}10^{10}$ VP/mL of influenza A virus.

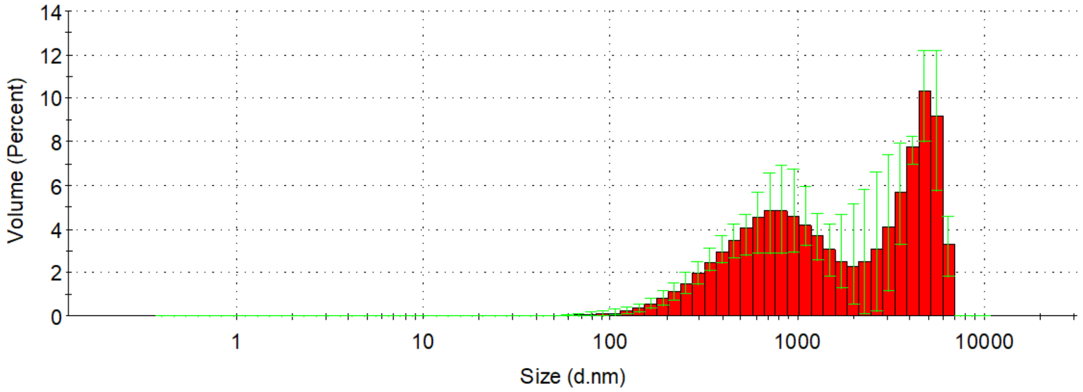

Figure S17. Dynamic light scattering. The volume distribution of silver nanoparticles in the sensor with the same amount of allantoic fluid as in $1.2{\cdot}10^{7}$ VP/mL of influenza A virus.

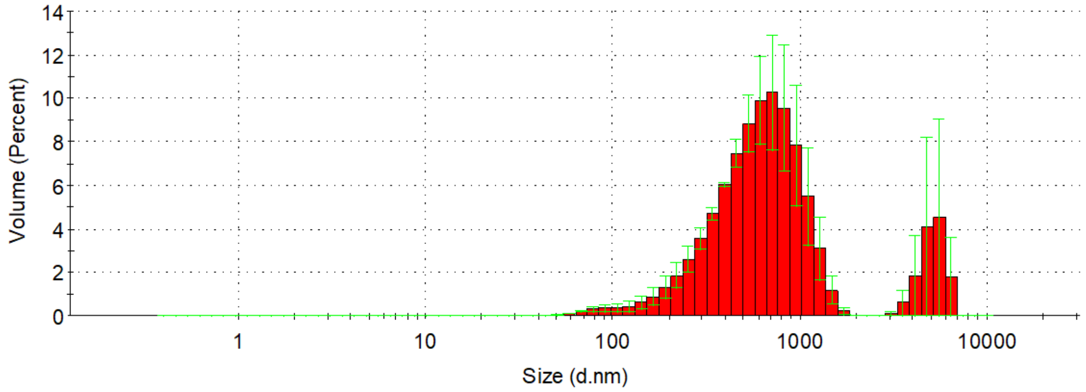

Figure S18. Dynamic light scattering. The volume distribution of silver nanoparticles in the sensor with the same amount of allantoic fluid as in $5 \cdot 10^7$ VP/mL of influenza A virus.

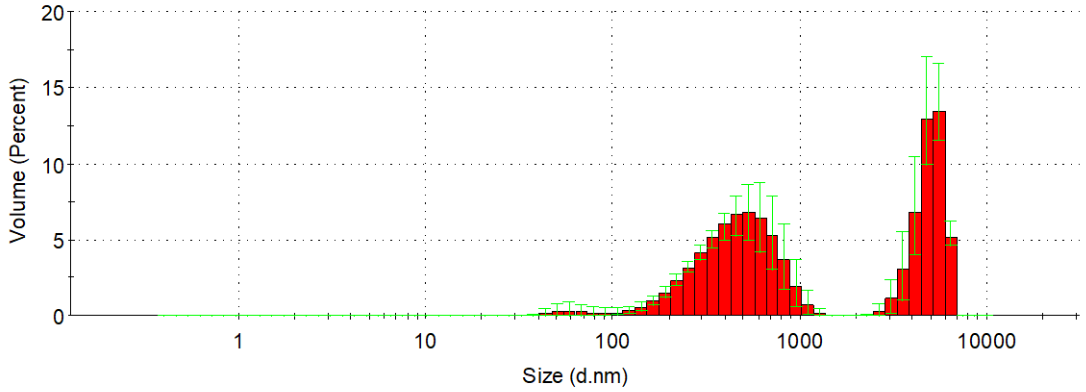

Figure S19. Dynamic light scattering. The volume distribution of silver nanoparticles in the sensor with the same amount of allantoic fluid as in $1.2 \cdot 10^8$ VP/mL of influenza A virus.

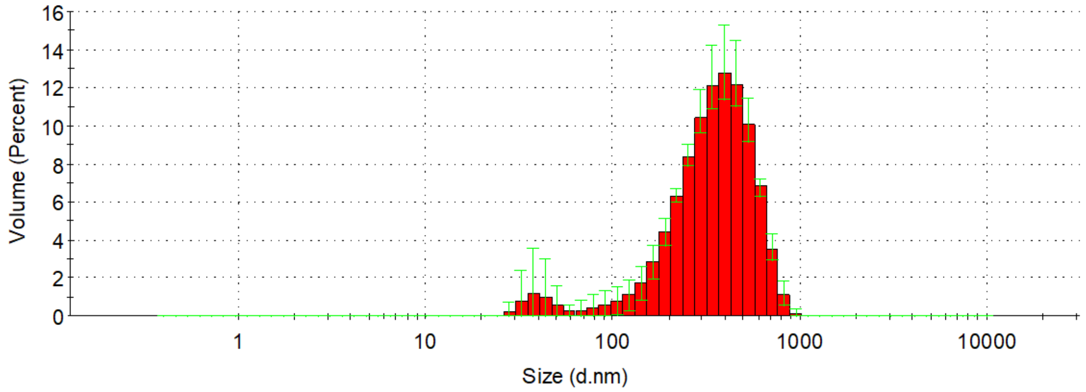

Figure S20. Dynamic light scattering. The volume distribution of silver nanoparticles in the sensor with the same amount of allantoic fluid as in $2.4 \cdot 10^8$ VP/mL of influenza A virus.

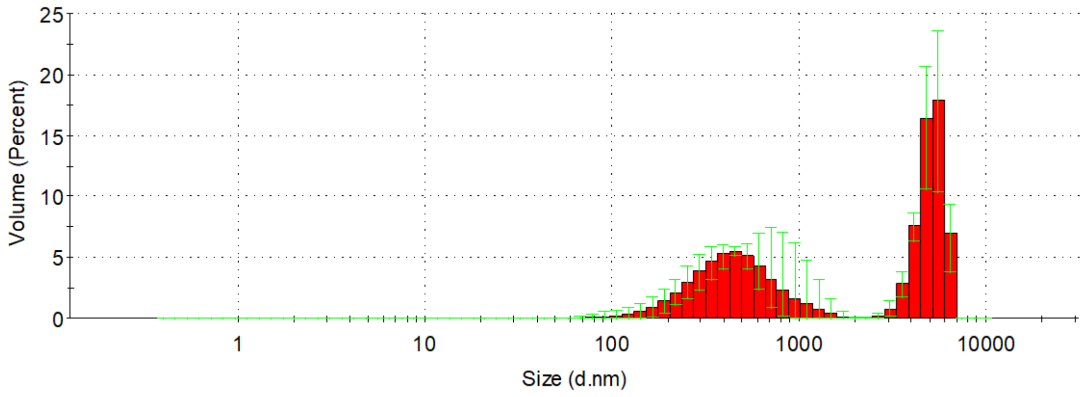

Figure S21. Dynamic light scattering. The volume distribution of silver nanoparticles in the sensor with the same amount of allantoic fluid as in 5·10^8 VP/mL of influenza A virus.

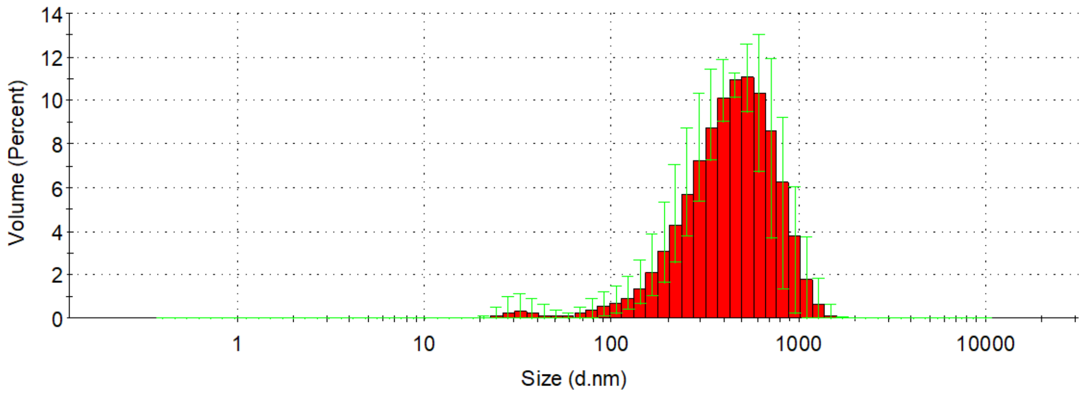

Figure S22. Dynamic light scattering. The volume distribution of silver nanoparticles in the sensor with the same amount of allantoic fluid as in 1.4·10^9 VP/mL of influenza A virus.

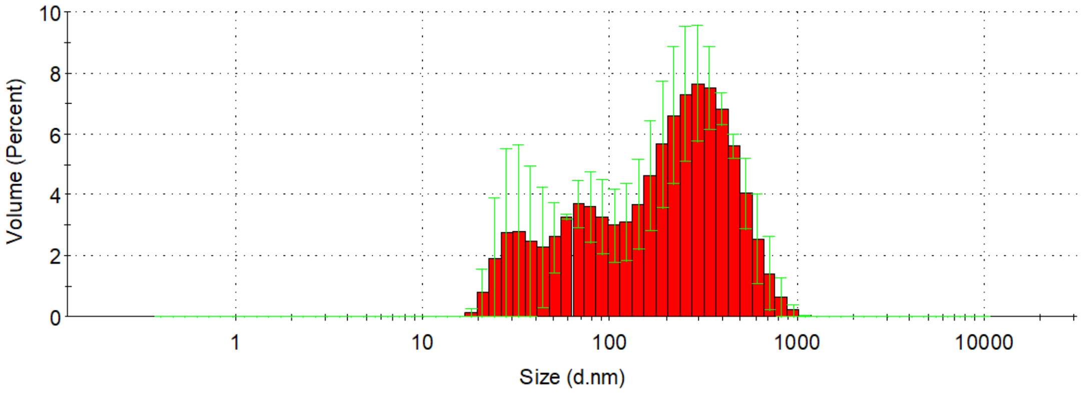

Figure S23. Dynamic light scattering. The volume distribution of silver nanoparticles in the sensor with the same amount of allantoic fluid as in 5·10^9 VP/mL of influenza A virus.

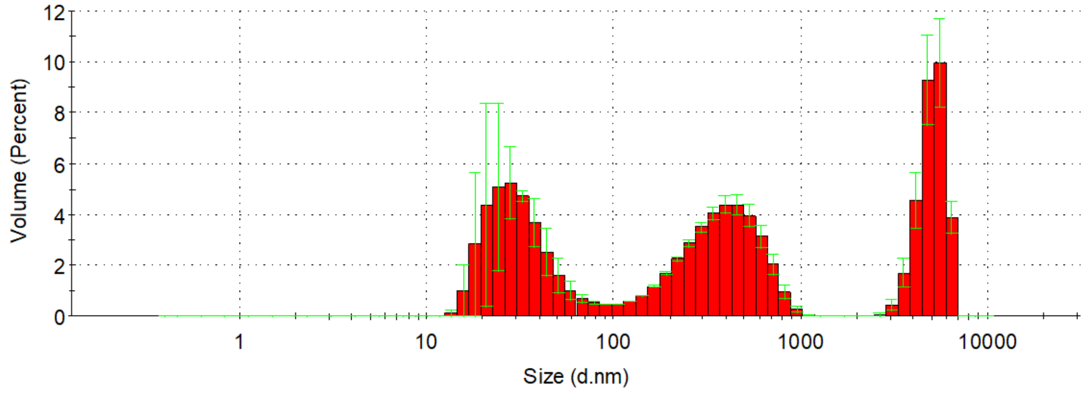

Figure S24. Dynamic light scattering. The volume distribution of silver nanoparticles in the sensor with the same amount of allantoic fluid as in 1·10^{10} VP/mL of influenza A virus.

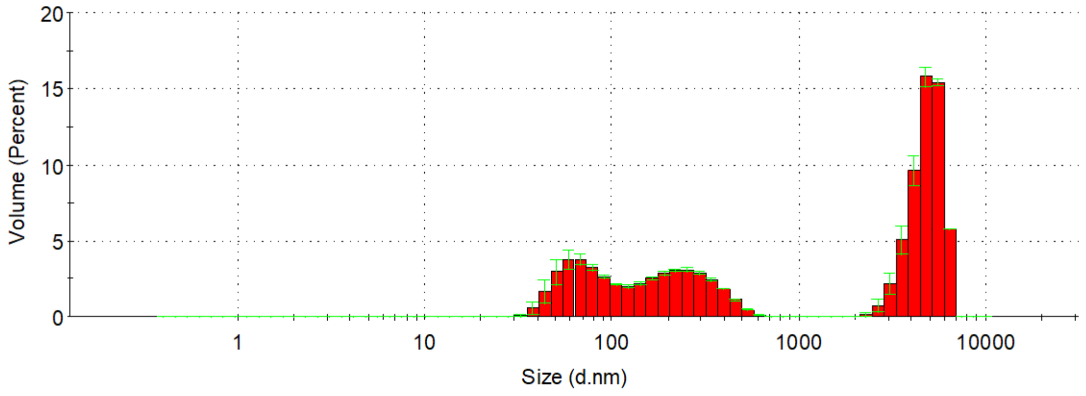

Figure S25. Dynamic light scattering. The volume distribution of silver nanoparticles in the sensor with the same amount of allantoic fluid as in 2·10^{10} VP/mL of influenza A virus.

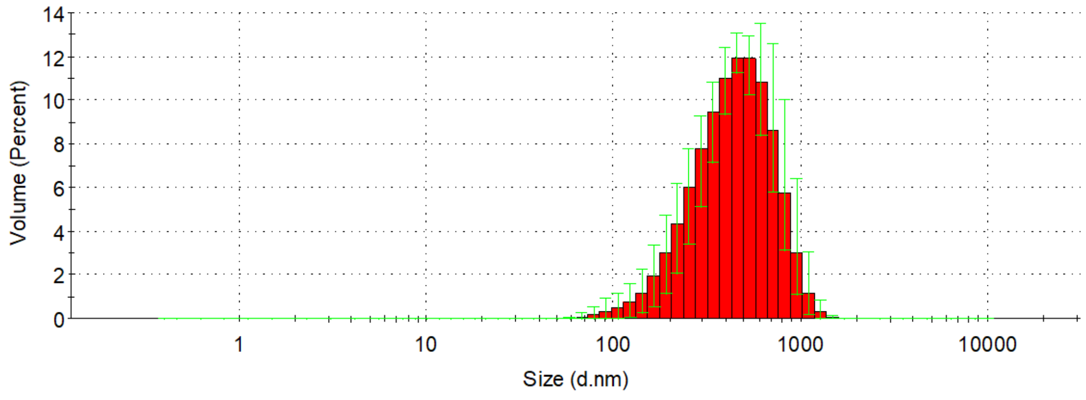

Figure S26. Dynamic light scattering. The volume distribution of silver nanoparticles in the sensor with 5·10^7 VP/mL of influenza B virus.

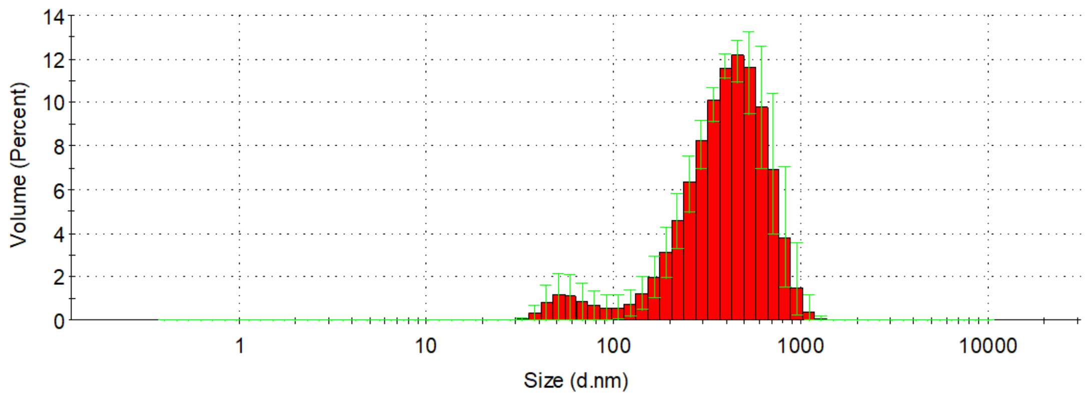

Figure S27. Dynamic light scattering. The volume distribution of silver nanoparticles in the sensor with $2{\cdot}10^8$ VP/mL of influenza B virus.

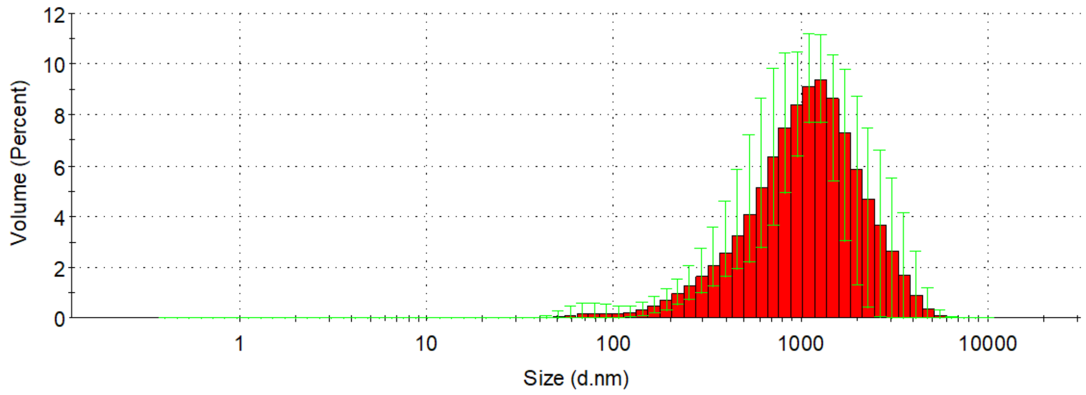

Figure S28. Dynamic light scattering. The volume distribution of silver nanoparticles in the sensor with $5{\cdot}10^8$ VP/mL of influenza B virus.

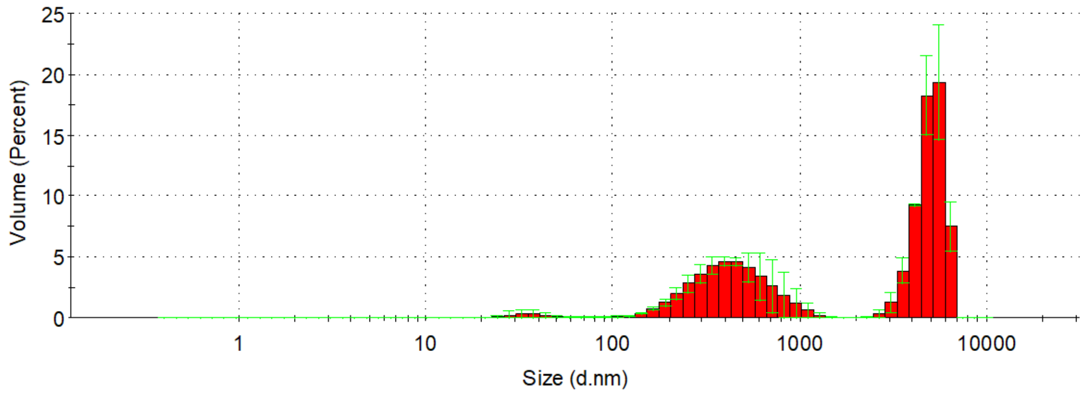

Figure S29. Dynamic light scattering. The volume distribution of silver nanoparticles in the sensor with $1{\cdot}10^9$ VP/mL of influenza B virus.

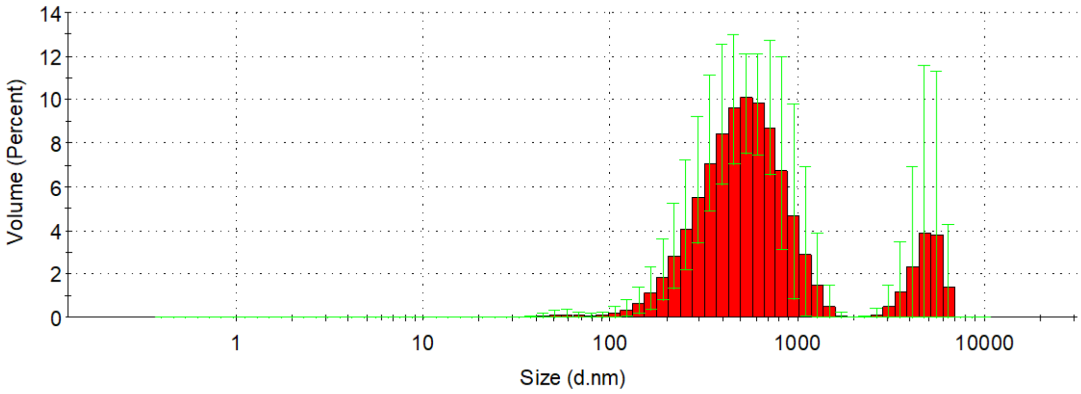

Figure S30. Dynamic light scattering. The volume distribution of silver nanoparticles in the sensor with $2{\cdot}10^9$ VP/mL of influenza B virus.

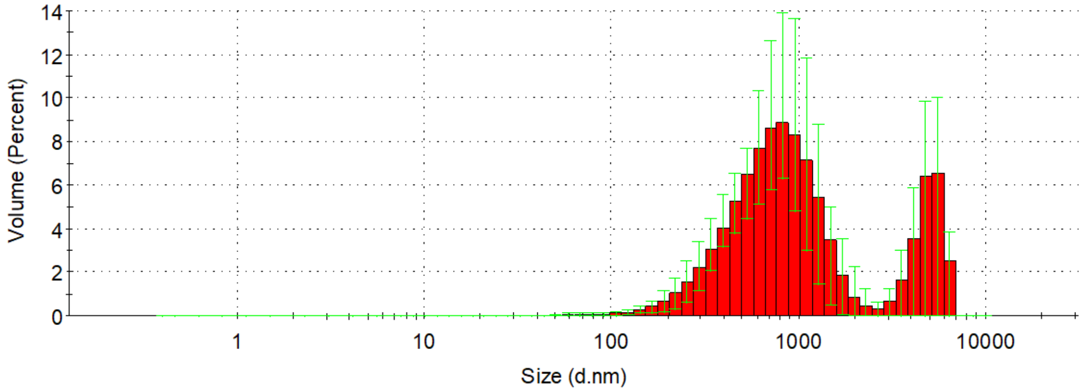

Figure S31. Dynamic light scattering. The volume distribution of silver nanoparticles in the sensor with $5{\cdot}10^9$ VP/mL of influenza B virus.

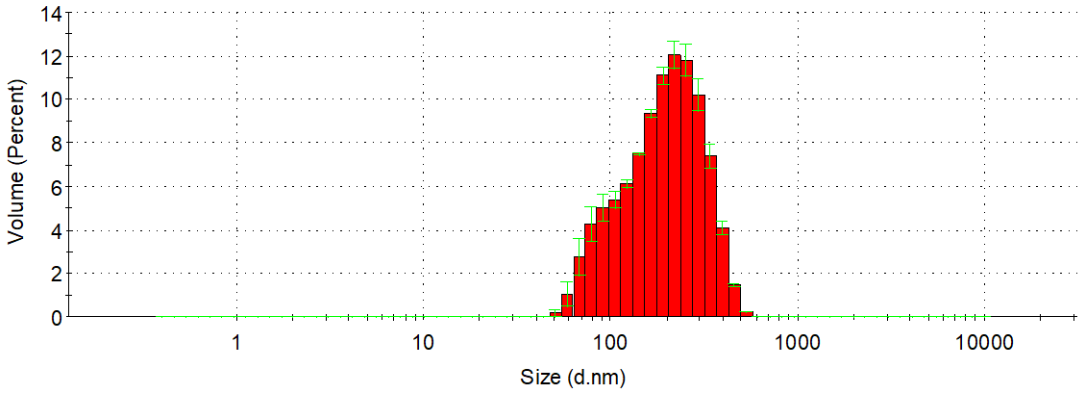

Figure S32. Dynamic light scattering. The volume distribution of silver nanoparticles in the sensor with $2{\cdot}10^{10}$ VP/mL of influenza B virus.

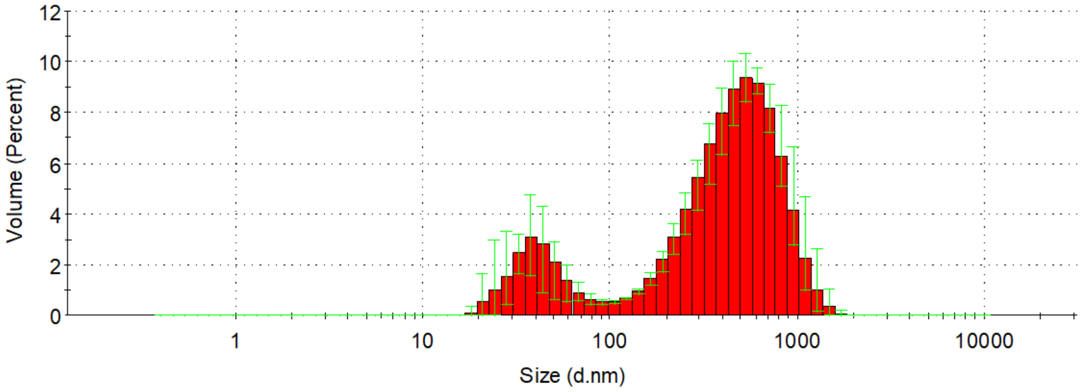

Figure S33. Dynamic light scattering. The volume distribution of silver nanoparticles in the sensor with $4{\cdot}10^{10}$ VP/mL of influenza B virus.

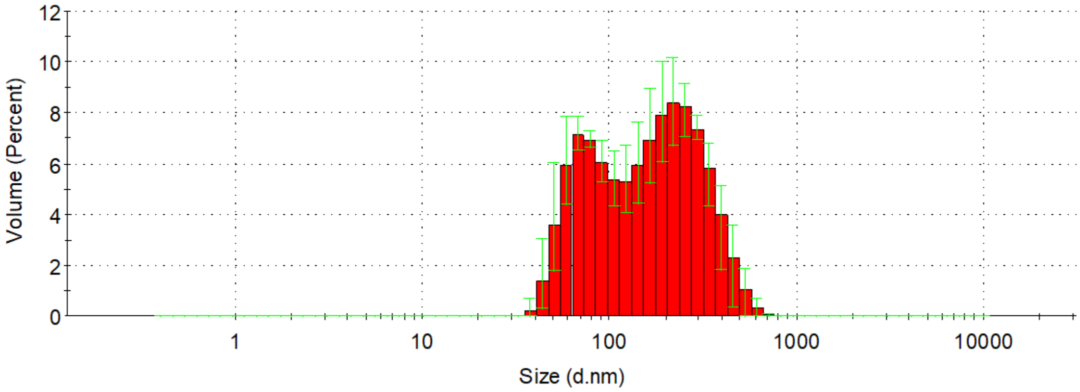

Figure S34. Dynamic light scattering. The volume distribution of silver nanoparticles in the sensor with $8{\cdot}10^{10}$ VP/mL of influenza B virus.

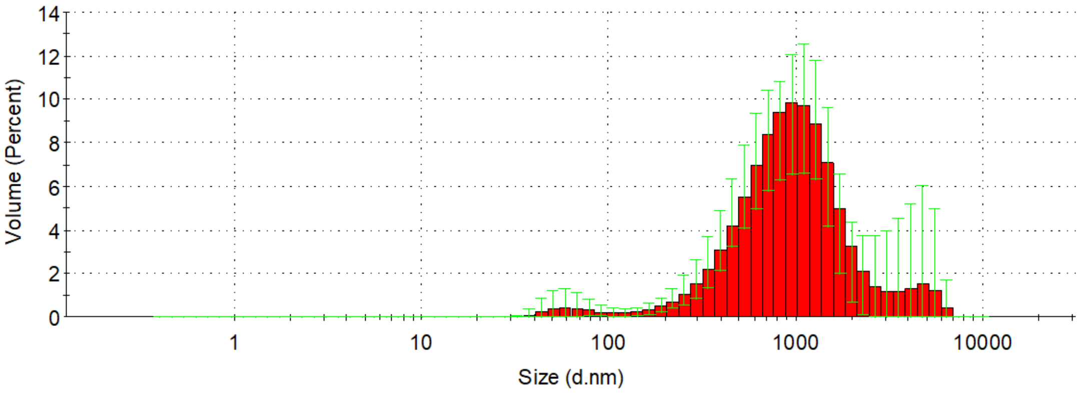

Figure S35. Dynamic light scattering. The volume distribution of silver nanoparticles in the sensor with $2{\cdot}10^{7}$ VP/mL of Newcastle disease virus.

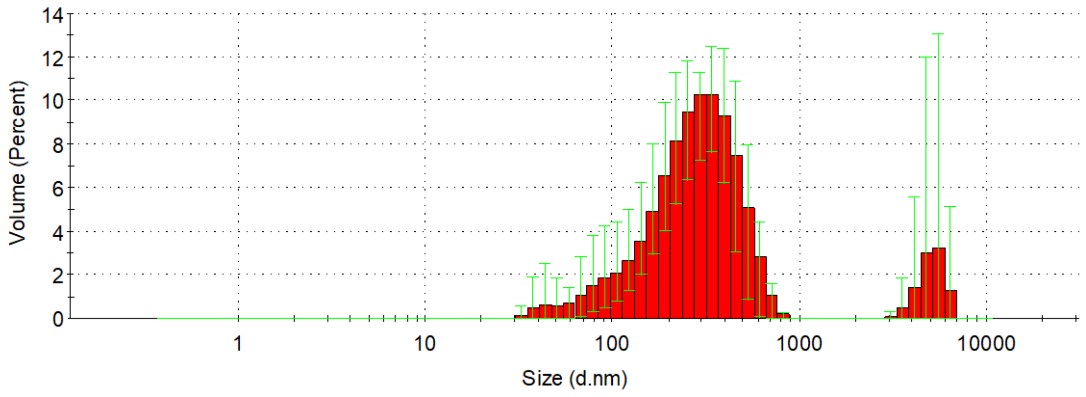

Figure S36. Dynamic light scattering. The volume distribution of silver nanoparticles in the sensor with $8 \cdot 10^7$ VP/mL of Newcastle disease virus.

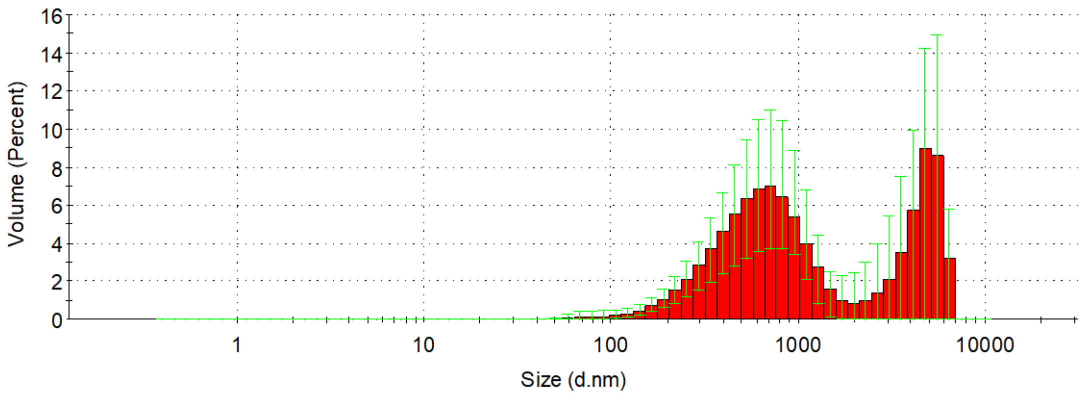

Figure S37. Dynamic light scattering. The volume distribution of silver nanoparticles in the sensor with $2 \cdot 10^8$ VP/mL of Newcastle disease virus.

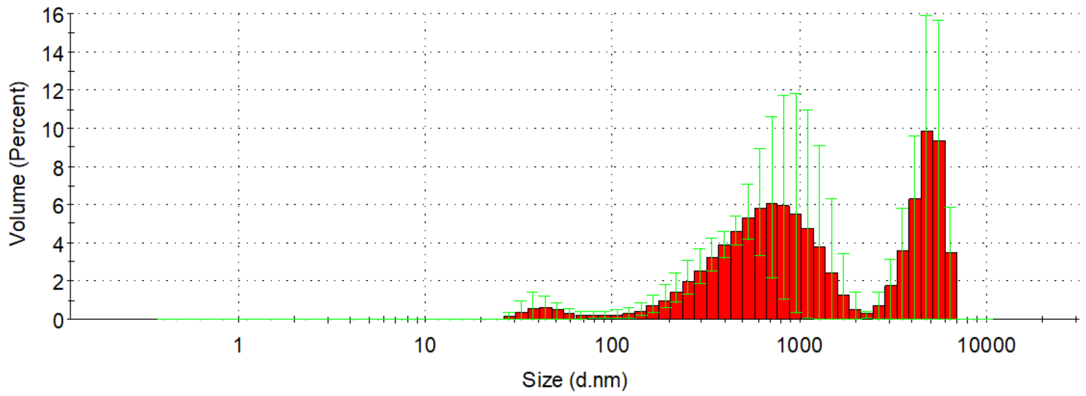

Figure S38. Dynamic light scattering. The volume distribution of silver nanoparticles in the sensor with $4 \cdot 10^8$ VP/mL of Newcastle disease virus.

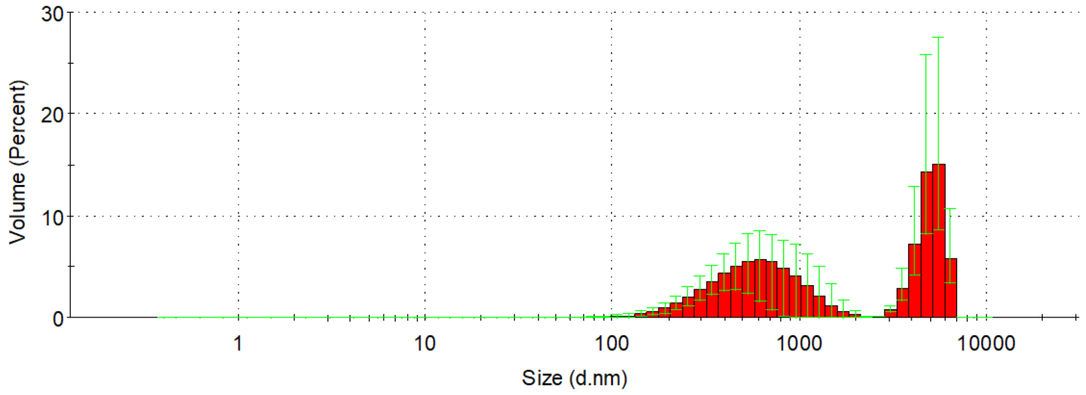

Figure S39. Dynamic light scattering. The volume distribution of silver nanoparticles in the sensor with $8{\cdot}10^8$ VP/mL of Newcastle disease virus.

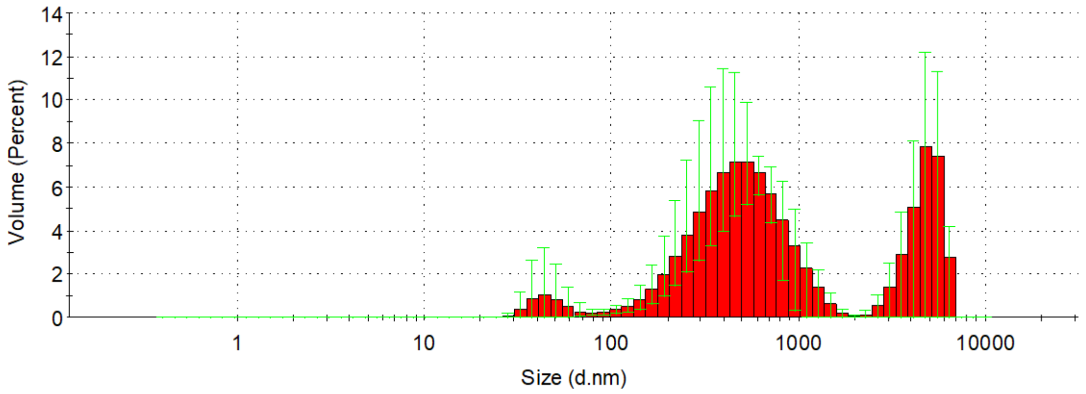

Figure S40. Dynamic light scattering. The volume distribution of silver nanoparticles in the sensor with $3{\cdot}10^9$ VP/mL of Newcastle disease virus.

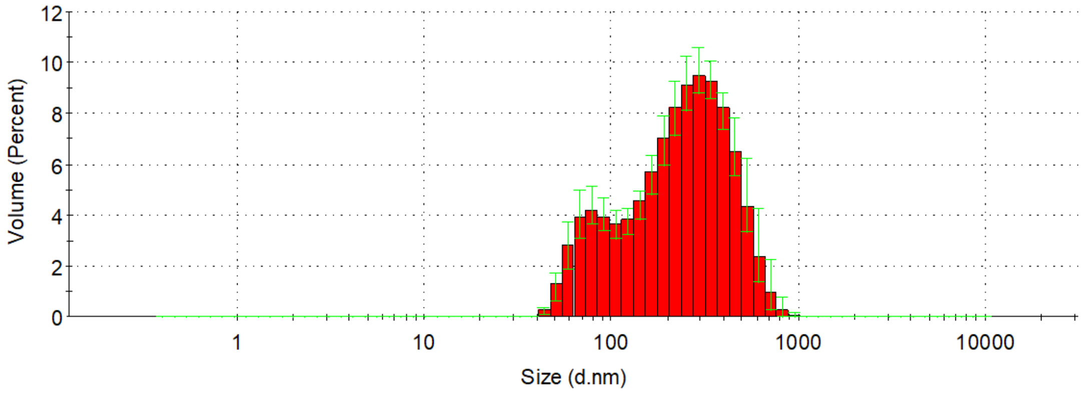

Figure S41. Dynamic light scattering. The volume distribution of silver nanoparticles in the sensor with $1{\cdot}10^{10}$ VP/mL of Newcastle disease virus.

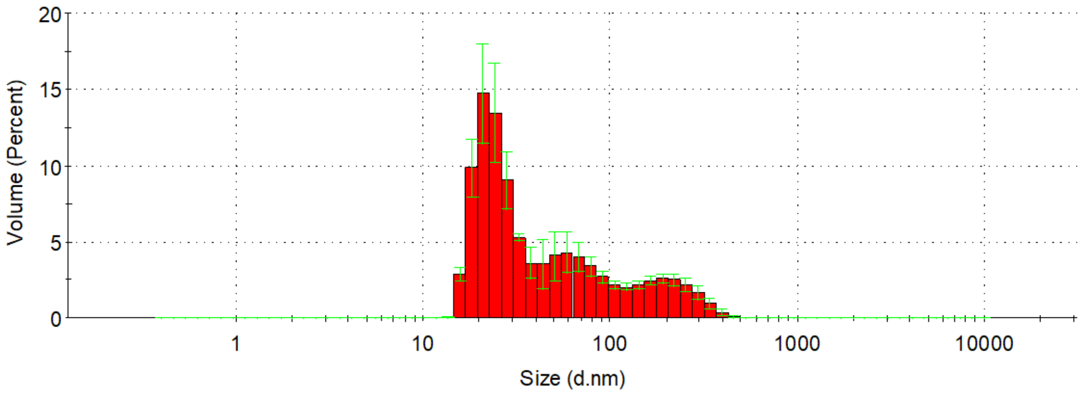

Figure S42. Dynamic light scattering. The volume distribution of silver nanoparticles in the sensor with $2{\cdot}10^{10}$ VP/mL of Newcastle disease virus.

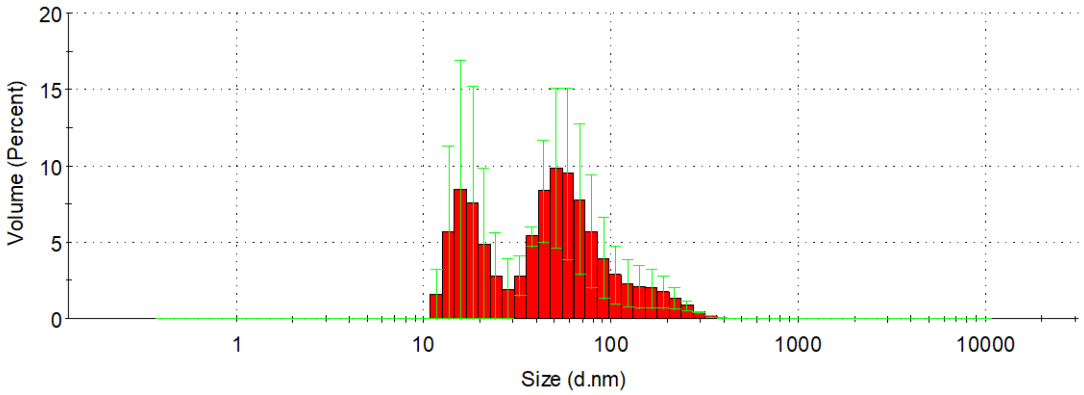

Figure S43. Dynamic light scattering. The volume distribution of silver nanoparticles in the sensor with $4{\cdot}10^{10}$ VP/mL of Newcastle disease virus.

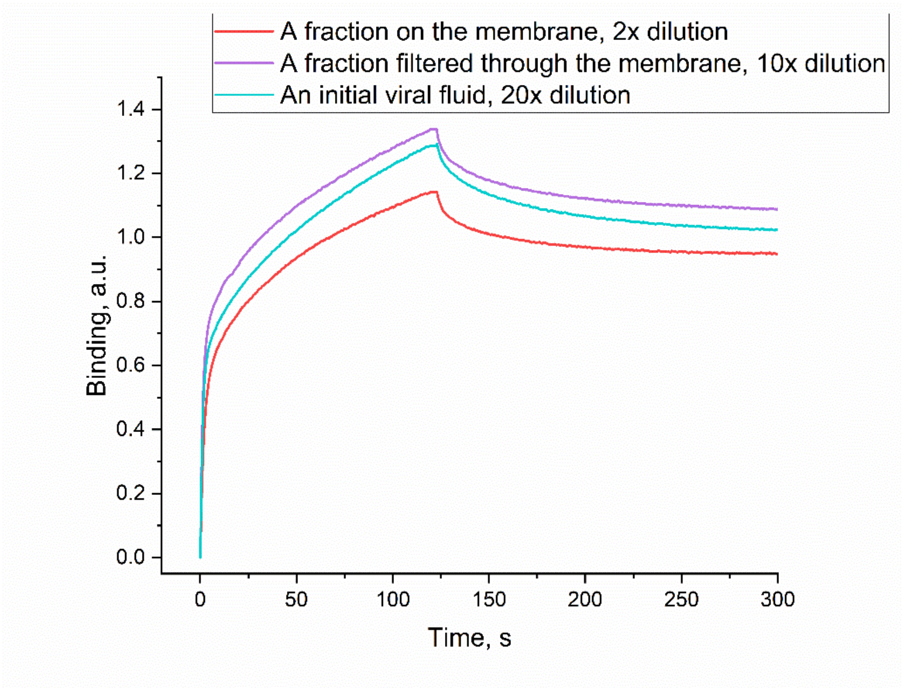

Figure S44. Binding of influenza A virus with different sample preparation to the aptamer immobilized on the sensor. The dilutions of the fluids were chosen to provide similar signals.

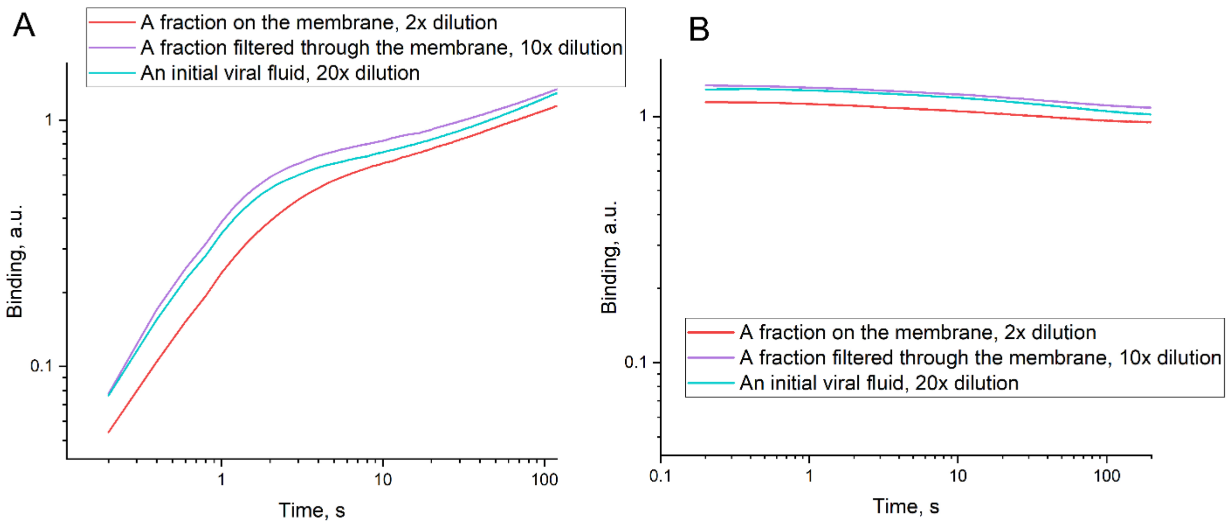

Figure S45. Binding of influenza A virus with different sample preparation to the aptamer immobilized on the sensor. The dilutions of the fluids were chosen to provide similar signals. The data are the same as in Figure S44 but represented in the logarithmic scale.

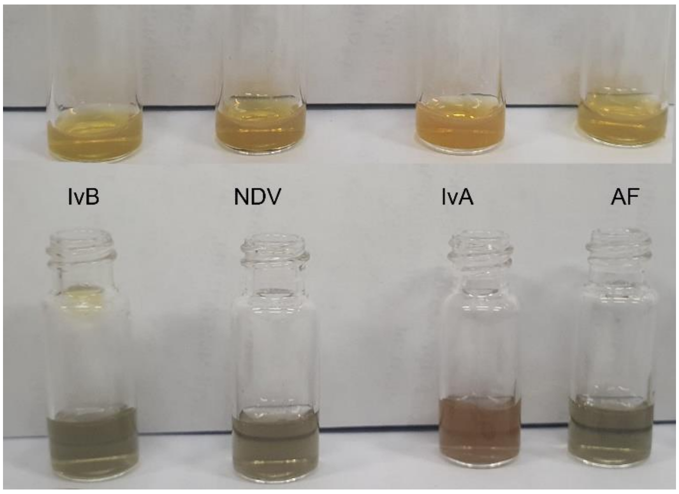

Figure S46. Color changes in $4 \cdot 10^{10}$ VP/mL solutions of viruses with silver nanoparticles with (lower row) or without (upper row) membrane filtration.
